# The development of community paramedicine; a restricted review

**DOI:** 10.1111/hsc.13985

**Published:** 2022-09-05

**Authors:** Brendan Shannon, Georgette Eaton, Chelsea Lanos, Matthew Leyenaar, Mike Nolan, Kelly‐Ann Bowles, Brett Williams, Peter O'Meara, Gary Wingrove, JD Heffern, Alan Batt

**Affiliations:** ^1^ Department of Paramedicine Monash University Frankston Victoria Australia; ^2^ Nuffield Department of Primary Care Health Sciences University of Oxford Oxford UK; ^3^ County of Renfrew Paramedic Service Pembroke Canada; ^4^ Department of Health and Wellness, Emergency Health Services Government of Prince Edward Island Prince Edward Island Canada; ^5^ International Roundtable on Community Paramedicine Duluth MN USA; ^6^ Indigenous Services Canada, Government of Canada Ottawa Ontario Canada

**Keywords:** community, community ine, mobile integrated health, paramedic, paramedic, paramedic, paramedic practitioner, restricted review

## Abstract

Community paramedic roles are expanding internationally, and no review of the literature could be found to guide services in the formation of community paramedicine programmes. For this reason, the aim of this restricted review was to explore and better understand the successes and learnings of community paramedic programmes across five domains being; education requirements, models of delivery, clinical governance and supervision, scope of roles and outcomes. This restricted review was conducted by searching four databases (CENTRAL, ERIC, EMBASE, MEDLINE and Google Scholar) as well as grey literature search from 2001 until 28/12/2021. After screening, 98 articles were included in the narrative synthesis. Most studies were from the USA (*n* = 37), followed by Canada (*n* = 29). Most studies reported on outcomes of community paramedicine programmes (*n* = 50), followed by models of delivery (*n* = 28). The findings of this review demonstrate a lack of research and understanding in the areas of education and scope of the role for community paramedics. The findings highlight a need to develop common approaches to education and scope of role while maintaining flexibility in addressing community needs. There was an observable lack of standardisation in the implementation of governance and supervision models, which may prevent community paramedicine from realising its full potential. The outcome measures reported show that there is evidence to support the implementation of community paramedicine into healthcare system design. Community paramedicine programmes result in a net reduction in acute healthcare utilisation, appear to be economically viable and result in positive patient outcomes with high patient satisfaction with care. There is a developing pool of evidence to many aspects of community paramedicine programmes. However, at this time, gaps in the literature prevent a definitive recommendation on the impact of community paramedicine programmes on healthcare system functionality.

**What is known about this topic ?**

- Community paramedicine is now widely implemented across much of Australasia, Canada, Finland, Ireland, the United Kingdom and the United States of America.
- The main drivers for the community paramedicine model have been the changing paramedic service caseloads that reflect aging populations and declining access to other health services.
- Community paramedicine models provide an opportunity for community paramedics to better meet the needs of disadvantaged communities.

**What this paper adds?**

- This review has highlighted that the education required for community paramedics, and the scope of the role they undertake, differs across continents, and across different jurisdictions within each country.
- There was a lack of standardisation in the implementation of governance and supervision models to support community paramedics in both their role and development.
- The inconsistency in outcomes reported in the literature demonstrate a gap in the current evidence.

## 1 INTRODUCTION

Of all the health disciplines that have progressed in the last decade, none have developed as significantly across the globe as paramedics. Emerging from a clinical environment that necessitated transport to hospital and definitive care, the profession has developed well beyond its initial boundaries of a transportation service. Perhaps one of the interesting things in this development of the profession is that it occurred similarly across the globe. Australasia (Andrew et al., 2020), Canada (Drennan et al., 2021), Finland (Keskimäki et al., 2019), Ireland (Cullinan et al., 2021), the United Kingdom (Claridge, 2021), and the United States of America (Mark et al., 2000) have all faced increasing demand in their respective health services over the last 5 years. Responding to this demand, services delivered by paramedics have also seen expansion, particularly those that result in treatment for patients in the community without hospital attendance.

Although paramedics may not have been an obvious choice for delivering community care due to their association with emergency response, it is their ability to adapt to any situation and patient complaint, that gives them the transferable skills required to assist all in the community at their time of need. With additional education, and increases in paramedic autonomy and scope of role (Eaton, Wong, et al., 2021), paramedics have excelled in their transition from a transport service to hospital to now assessing and managing patients in the community.

Although community paramedic roles are expanding internationally, no review of the literature could be found to guide services in the formation of community paramedicine programmes. For this reason, the aim of this restricted review was to explore and better understand the successes and learnings of community paramedic programmes. We sought to review the published research (both peer‐reviewed and grey literature) on the following topics as it relates to community paramedicine:

- Education requirements
- Models of delivery
- Clinical governance, supervision and other structural supports
- Scope of community paramedicine roles
- Outcomes associated with community paramedic programmes

## 2 METHODS

Using restricted review (sometimes referred to as a rapid review) methodology (Garritty et al., 2021), we used the population, concept, context (PCC) approach to draft research questions for the restricted review. The review protocol was registered on the Open Science Framework in December 2021 (https://osf.io/qxwes/).

### 2.1 Search strategy

We used an existing validated review search strategy by Eaton et al. (2020) (see Appendix A), searching electronic databases CENTRAL [2001–28/12/2021], ERIC (ProQuest) [2001–28/12/2021], EMBASE (OvidSP) [2001–28/12/2021], MEDLINE (OvidSP) [2001–28/12/2021] and Google Scholar [2001–28/12/2021]. Keywords and subject headings were adapted as required for individual databases. The CADTH Grey Matters toolkit was used to guide grey literature searching (Canadian Agency for Drugs Technologies in Health, 2015). Citation chaining of final included studies was conducted via “*citationchaser*” software (Haddaway et al., 2021).

### 2.2 Eligibility criteria

Articles of any study design that discussed community paramedicine programmes (including mixed‐response models whereby paramedics collaborate with or work alongside other healthcare professionals) were included. We excluded literature that discussed programmes that did not meet the definition of community paramedicine (e.g. ambulance‐based retrieval services, home visits by nursing or general practitioners) and conference abstracts. In addition, we excluded case studies and commentary pieces where no community paramedicine programme was studied, as well as magazine articles and news reports from the grey literature. Papers not available in English were excluded.

### 2.3 Study selection

We imported all search results into Covidence systematic review management software (Babineau, 2014) where duplicates were removed. Titles and abstracts were screened independently by two reviewers (BS, AB, GE, CL or MN) for exclusion using the eligibility criteria. This was followed by a full‐text review of the remaining articles by two reviewers (BS, GE, GW or MN) using the same eligibility criteria. Conflicts were resolved by discussion or involvement of a third reviewer (BS, AB or CL).

### 2.4 Data extraction

We designed and used a data extraction form informed by the Cochrane Handbook for Systematic Reviews (Lasserson et al., 2019). The form was piloted and updated until the research team reached consensus on the final version. Data were extracted by multiple reviewers (BS, AB, GE, ML, MN, CL, JH, GW). Informed by restricted review methodology (Plüddemann et al., 2018), a random 20% sample (*n* = 19) was audited for verification by a second author (BS).

### 2.5 Synthesis and analysis

Synthesis involved a content analysis of included studies focusing on education; models of delivery; clinical governance, supervision and other structural supports; scope of role; and outcomes from community paramedicine programmes.

### 2.6 Quality assessment

A risk‐of‐bias appraisal of included peer‐reviewed literature using the Mixed Methods Appraisal Tool (MMAT) was conducted (Hong et al., 2018). One reviewer assessed the risk‐of‐bias of the included studies, and this was verified by a random 20% sample audit by a second author (BS). Risk‐of‐bias appraisal was limited to the primary outcome measure for each study (Garritty et al., 2021).

## 3 RESULTS

### 3.1 Search results and study selection

**FIGURE 1:**
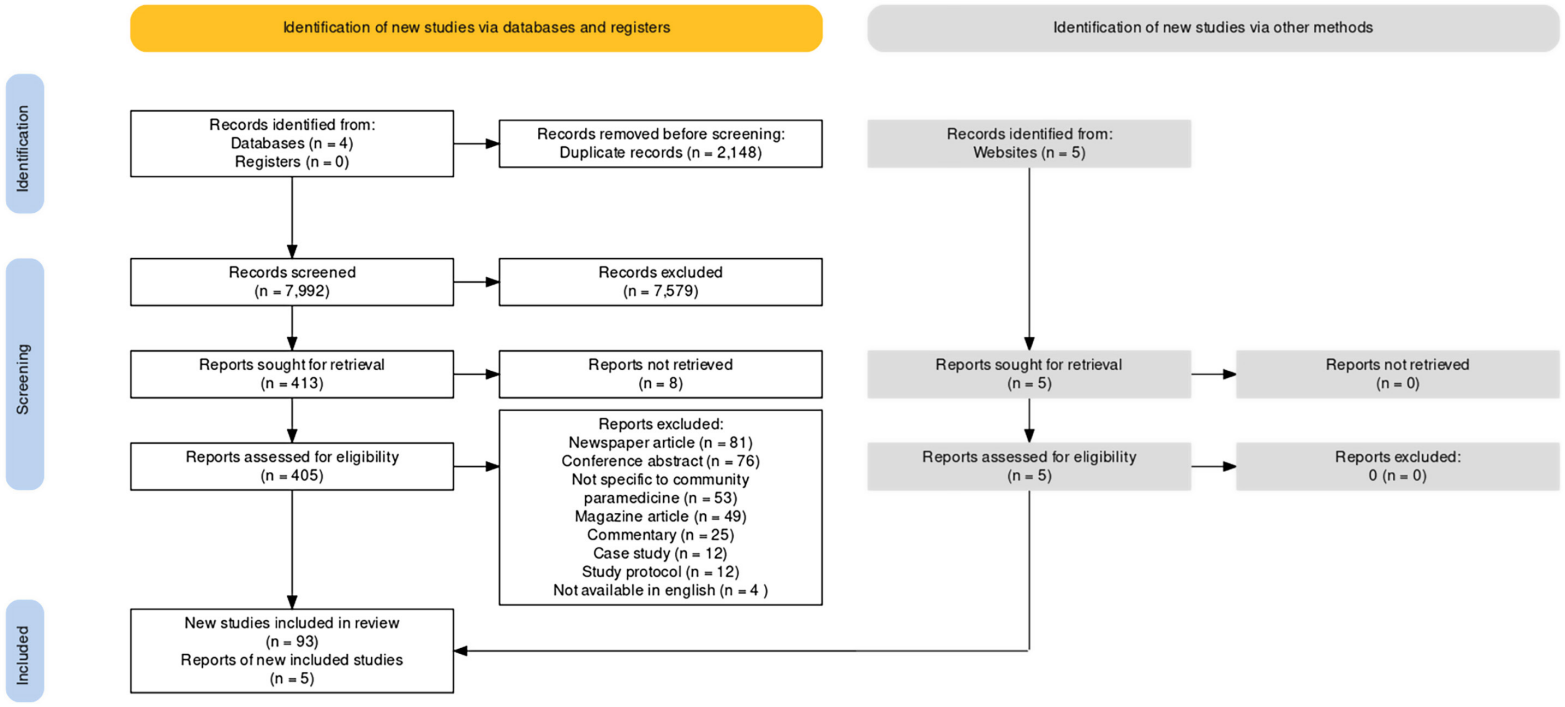
Prisma flow diagram.

The initial search strategy and citation chaining yielded 10,130 citations for screening. We identified an additional five citations through searches of grey literature. After elimination of duplicates (2148), we screened 7992 studies at the title and abstract level (Level 1). This led to the exclusion of 7579 citations. After performing a full‐text review of 410 studies, 98 studies were included for extraction and analysis. Figure 1 illustrates a PRISMA flow diagram of these findings, and Appendix B lists the included studies.

### 3.2 Characteristics of included studies

**FIGURE 2:**
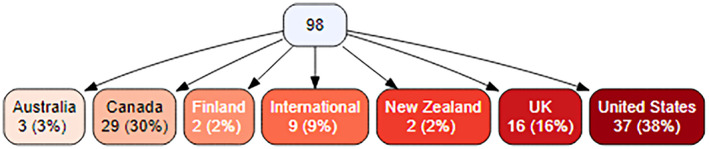
Geographical origin of studies included in this review.

Included studies were published between 2003 and 2021, with the majority published from 2016 onwards (69 of 98 studies). Most were published as peer‐reviewed articles (*n* = 93), with the remainder being reports (*n* = 5). Most studies were from the United States (*n* = 37, 38%), followed by Canada (*n* = 29, 30%) and the United Kingdom (*n* = 16, 16%) (Figure 2).

The most common methodologies involved qualitative approaches to data collection and analysis (*n* = 21), followed by systematic reviews (*n* = 13), cohort studies (*n* = 8) and randomised controlled trials (*n* = 6). Populations studied included service users (with varying medical/social needs and histories), paramedics and community paramedics, other healthcare professionals, health system managers and community members (e.g. relatives and carers). Sample sizes ranged from six (Proctor, 2019) to 43,856 (Leyenaar, McLeod, et al., 2021).

**FIGURE 3:**
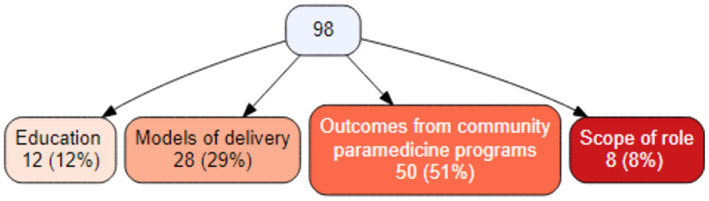
Primary category of evidence investigated by studies included in this review.

Most studies reported on outcomes of community paramedicine programmes (*n* = 50, including quality of life, patient satisfaction and economic impacts), followed by models of delivery (*n* = 28, including clinical governance, supervision and other structural supports). Several studies reported on more than one of the descriptive categories (Figure 3).

### 3.3 Quality assessments results

Quality assessment using the MMAT was possible in 68 of 98 studies. No studies included in the review warranted exclusion based on a significant risk of bias identified in quality assessment; however, lower levels of evidence/study design were most common (Burns et al., 2011). The most common concern on assessment was due to poorly or vaguely described methods being reported. Of the 30 studies without assessment using the MMAT (where no relevant category exists) there was deemed to be no significant risk of bias in methods, and this most commonly was with literature reviews. See Appendix C for tabulated quality assessment results.

### 3.4 Education

Twelve studies reported on the education of community paramedics, the competencies required of community paramedics and/or the gaps that exist in service delivery and the future directions of education to provide such services.

#### 3.4.1 Community paramedicine education requirements

The descriptions of community paramedic education included various forms of in‐service education (Chan et al., 2019; Ruest et al., 2017), community‐college based curricula and programmes (Choi et al., 2016; Pearson et al., 2014) and postgraduate level degrees (Chan et al., 2019; Eaton et al., 2020; Eaton, Happs, & Tanner, 2021; Eaton, Wong, et al., 2021). A systematic review by Chan et al. (2019) reported a median training time of 240 h per trainee per programme with a range of 3.5 to 2080 h, This is similar to the US National Curriculum and Career Pathway for Community Paramedicine (Paramedic Health Solutions, 2016) which outlined that community paramedics must complete a 300‐h education programme that includes primary care clinical rotations. This is the only curriculum reported in the literature (Boykin et al., 2018). A realist review by Eaton, Wong, et al. (2021) reported a master's degree as the most common educational requirement for community paramedics.

#### 3.4.2 Competencies required of community paramedics

Only one study (Ruest et al., 2017) described a process whereby a newly implemented programme evaluated paramedics against a set of desirable competencies (knowledge, skills and attitudes—KSAs) informed by the National Occupational Competency Profile for Paramedics (Paramedic Association of Canada, 2011).

### 3.5 Models of service delivery

A total of 28 studies reported on models of service delivery. In addition to outlining the service delivery models, the studies discussed the need for programmes to be designed around community needs, specific innovations in response to COVID‐19, challenges facing service delivery and future directions for research and knowledge sharing.

#### 3.5.1 Service delivery models

A variety of models of service delivery were reported in the literature, and these are broadly described in Table 1, informed by Leyenaar, Strum, and Haque (2019). According to Leyenaar, McLeod, et al. (2019) 75% of Ontario's municipal paramedic services delivered more than one community paramedicine model of care, serving an estimated population of 56,640, while referring 23,040 service users to other services. O'Meara et al. (2016) outlined that despite how the model was delivered, community paramedicine differs from other paramedic service delivery by engaging with communities; through its situated practice; collaboration with primary healthcare; integration with health, aged care and social services; focused governance and leadership; embrace of higher education; and providing treatment and transport options. From a service delivery perspective, this highlights the importance of integration with the healthcare system. Community paramedics carried out fewer investigations, provided more treatments and were more likely to discharge patients home than the usual providers. In addition, through working in different settings across traditional professional boundaries, community paramedics impacted how services were delivered locally (Mason, Knowles, et al., 2007).

**TABLE 1:** Community paramedicine service delivery models

Model	Description
Community assessment and referral	Community paramedics connect individuals with other care providers, including community care services
Community paramedic‐led clinics	Community paramedics advertise and promote health promotion and preventative care services (including influenza vaccination, chronic disease education, blood pressure checks)
Home visit programmes	Community paramedics work with other healthcare services to maximise “at‐home” services for those who repeatedly call or are at risk of frequent 911 utilisation due to medical conditions and/or unmet social needs
Remote patient monitoring programmes	Community paramedics work with primary care providers to address issues proactively via 24‐hour home‐based monitoring programmes for chronic health conditions such as COPD, CHF and diabetes
Community paramedic specialist response	Community paramedics work closely with 911 colleagues in a coordinated and cooperative manner to enable access to other healthcare providers
Hospital discharge/transitional care support	Community paramedic programmes partner with hospitals to facilitate improved timeliness of discharge from hospital, with follow‐up by community paramedics
Mental health and addictions support	Community paramedics are part of mental health crisis response teams, provide care in homeless shelter programmes, and assist in medical care provision and oversight at safe consumption and treatment sites
Palliative care support	Community paramedics provide care for palliative care patients at home aligned with care preferences of those receiving care
Influenza surge programmes	Community paramedics work with at‐risk populations to increase vaccination rates and manage influenza‐like presentations in retirement, nursing and other residential environments
COVID response programmes	Community paramedicine programmes provided COVID response activities including testing, vaccination clinics and logistical support for public health partners

#### 3.5.2 Community need assessment

A key recommendation and lesson reported in the literature across multiple studies was the essential role of understanding the community needs and factors that enabled a sustainable community paramedicine programme (Leyenaar, McLeod, et al., 2019; Pearson & Shaler, 2017; Seidl et al., 2021). O'Meara et al. (2015) advised that engaging appropriately with the community can result in more integrated paramedic services, working as part of a less‐fragmented system across the health, aged care and social service sectors. This was found to also be important to prevent duplication and overlap of existing service delivery (Feldman et al., 2021).

#### 3.5.3 Service delivery challenges

Programmes outlined three principal challenges: funding, data‐sharing and reporting, and regulatory issues (Batt, Hultink, Lanos, et al., 2021; Leyenaar, McLeod, et al., 2019; Martin & O'Meara, 2019; Pearson & Shaler, 2017). Cooper et al. (2007) suggested that further work was required to evaluate the development of practice, the quality of care and the cost benefits of community paramedicine, which is echoed in other papers (Rasku et al., 2019; Tangherlini et al., 2016). Evaluation of community paramedicine programmes has increased in recent years and is explored later in this manuscript. A lack of guidance and inconsistent interpretation regarding programmes and scope of practice was also highlighted (Glenn et al., 2018), while Batt, Hultink, Lanos, et al. (2021) recommended the establishment of appropriate quality indicators for community paramedicine (i.e., not traditional ambulance service quality indicators).

### 3.6 Governance and clinical support

Twenty‐one studies from the 28 categorised under models of service delivery (see figure 3) reported on clinical governance, supervision or medical oversight and other structural supports, including collaboration with other healthcare staff.

#### 3.6.1 Integrated interdisciplinary collaboration

Several models existed where community paramedics were integrated alongside other professional groups. Whalen et al. (2018) reports a combined nurse and paramedic team offering overnight urgent and emergency care, where the unique professional identities of both groups enabled a more holistic patient experience. A more recent model used community paramedics as a delegated home visit response from the heart failure specialist (Feldman et al., 2021), to expedite patient access to non‐emergency treatment associated with their condition. Similarly, Boykin et al. (2018) outlined a similar model involving paramedics, pharmacists and advanced practitioners in cardiology responding to patients with heart failure. Other programmes included community paramedics working in Mental Health Crisis Response Teams (Leyenaar, McLeod, et al., 2021) providing care in homeless shelter programmes (Tangherlini et al., 2016) and working within a rapid access team alongside Family Physicians (Barrett et al., 2004). The need for community paramedics to have effective clinical and managerial links with other health providers was well documented (Flint, 2019; Mason, Knowles, et al., 2007; O'Meara et al., 2015; Rasku et al., 2019; Whalen et al., 2018). The development of interagency links across different services also enabled the development of patient referral processes (Barrett et al., 2004). However, the key to integration were governance structures to support role implementation and reduce duplication between professional groups (Leyenaar et al., 2018; Seidl et al., 2021).

#### 3.6.2 Medical oversight

Although evidence suggested that community paramedics, for the most part, worked without direct medical supervision, 10 studies outlined the provision of remote medical oversight or physician support (Abrashkin et al., 2019; Feldman et al., 2021; Flint, 2019; Gingold et al., 2021; Glenn et al., 2018; Leyenaar et al., 2018; O'Meara et al., 2015; Seidl et al., 2021; Tangherlini et al., 2016; Whalen et al., 2018). In a majority of studies, it was not clear whether medical oversight directed the provision of patient care or was in place as a clinical support to troubleshoot clinical problems. Pearson and Shaler (2017) found that in jurisdictions where there was a lack of legislation underpinning professional roles, more robust clinical supervision was required. Leyenaar et al. (2018), however, in their review found almost exclusively use of clinical on‐call support for assistance, rather than permission to treat. There are also arguments that although there are valid reasons to support high levels of medical supervision for community paramedics, this should not be at the deficit of developing the professional practice of community paramedics (O'Meara, 2003; O'Meara et al., 2015).

#### 3.6.3 Standard operating procedures

The need for governance structures to support the integration and utilisation of community paramedics was a common occurrence (Flint, 2019; Glenn et al., 2018; Mason, Knowles, et al., 2007; Pearson & Shaler, 2017; Rasku et al., 2019; Seidl et al., 2021). As well as linking legislation to clinical supervision, Pearson and Shaler (2017) found that stronger standard operating procedures and protocols existed in jurisdictions with a lack of legislation to support professional conduct. However, there was evidence that while protocols existed, they were not always followed as they did not cover all components of the community paramedicine role, such as social assessments (Seidl et al., 2021).

### 3.7 Scope of role

Eight studies reported on the scope of the role of community paramedics. Across the literature reviewed, there was no clear standardisation regarding what an expanded scope of role for community paramedics would include, especially since practice guidelines were still being established as paramedics move to work in these relatively new clinical areas (Eaton, Wong, et al., 2021; Leyenaar, Allana, et al., 2021). However, three key components that were considered to be a staple requirement of the scope of the role expected of a community paramedic where general health assessment, psychosocial assessment and health promotion.

#### 3.7.1 General health assessment

All studies (Evans et al., 2014; Ford‐Jones & Daly, 2020; Keefe et al., 2020; Leyenaar, Allana, et al., 2021; Leyenaar, McLeod, et al., 2019; Stirling et al., 2007; Xi et al., 2021) outlined the importance for community paramedics to be able to undertake a general health assessment. Eight years ago, Evans et al. (2014), found that a general health assessment was among one of the most common roles reported in their literature review, with an emphasis on acute minor conditions, rather than complex multi‐organ disease. Similar results have been noted more recently in their cross‐sectional environmental scan, Leyenaar, McLeod, et al. (2019) found that all community paramedics undertook a generic health assessment across all organ systems. However, a multisystem assessment was less common, with no community paramedics undertaking an assessment featuring the neuromusculoskeletal system, for example. A more recent literature review outlines that the generalist training given to paramedics prepares them well for the management of a wide spectrum of undifferentiated illnesses, for which a general health assessment would be required (Xi et al., 2021).

#### 3.7.2 Psychosocial assessment

The assessment and management of behavioural health emergencies as a routine part of ambulance work were considered by Keefe et al. (2020) to be a transferable trait into community paramedicine. Slightly more recently, a Delphi study in Canada has outlined that the ability for community paramedics to undertake a psychosocial assessment is likely due to the absence of time‐sensitive situations in which paramedics conduct their assessments (Leyenaar, Allana, et al., 2021). Evans et al. (2014) outlined that this lack of time–pressure enables community paramedics to undertake safeguarding and risk assessments while in patients homes. However, another paper found that although assessment for psychosocial needs may be part of the extended role commensurate with community paramedicine, this was one aspect that would not necessarily form part of a paramedics initial training (Ford‐Jones & Daly, 2020). Indeed, Keefe et al. (2020) also found that experiential learning played a key part in the ability of community paramedics to expand the scope of their role to undertake a more holistic assessment that included biopsychosocial health.

#### 3.7.3 Health promotion

The expanded scope of role for paramedics was also considered to include health assessment and prevention of ill‐health (Evans et al., 2014; Leyenaar, Allana, et al., 2021; Stirling et al., 2007). In rural Australia, an expanded scope of role was found to enable community engagement with local health services (Stirling et al., 2007), where health promotion and illness prevention had a meaningful impact at the community level. Leyenaar, Allana, et al. (2021) found that through their engagement and general health assessment, community paramedics were likely to identify medium‐term and long‐ term care needs. Indeed, Evans et al. (2014) consider that the holistic nature of the assessment may be particularly suited to assess the health requirements of those aged over 60.

### 3.8 Outcomes associated with community paramedicine programmes

Outcomes of community paramedicine programmes were reported in 50 studies. The outcomes associated with community paramedicine programmes were categorised into five outcome categories:

- Impact on emergency calls, rates of transportation and hospital admissions
- Economic outcomes
- Patient health outcomes
- Patient satisfaction
- Community paramedic satisfaction and qualitative insights into the role

### 3.9 Impact on emergency calls, rates of transportation and hospital admissions

The most common outcome measure to evaluate community paramedicine programmes was the impact on rates of transportation and presentations to the Emergency Department (ED), impact on rates of Emergency Medical Services (EMS) calls, and admissions to hospital. 20 studies (Agarwal et al., 2019; Bennett et al., 2018; Brydges et al., 2016; Castillo et al., 2016; Choi et al., 2016; Cooper et al., 2007; Dixon et al., 2009; Gingold et al., 2021; Halter et al., 2006; Hänninen et al., 2020; Jensen et al., 2015; Leduc et al., 2021; Leyenaar, McLeod, et al., 2019; Mason et al., 2008; Mason, O'Keeffe, et al., 2007; Misra‐Hebert et al., 2021; Nejtek et al., 2017; Quatman‐Yates et al., 2021; Ruest et al., 2012; Tangherlini et al., 2016; Thompson et al., 2014) investigated community paramedicine across these outcome measures, with the impact on ED presentations the most frequently reported outcome measure. See Table S1 for tabulated results.

#### 3.9.1 Impact on emergency department presentations

Community paramedicine programmes were found to influence the net reduction of ED visits compared with routine pathways of care. In studies that compared the effect of emergency department visits compared with a control group not receiving community paramedicine intervention the reduction in ED presentations ranged from 21% (Castillo et al., 2016) to 58.7% (Bennett et al., 2018). Comparison control groups were found to have an increase in ED visits while the intervention of community paramedicine programmes provided a net overall reduction. Compared with control group participants, community paramedicine participants were found to be less likely to attend the ED (relative risk [RR] 0.72, 95% CI = 0.68 to 0.75) (Mason et al., 2008), and there was also reported to be a reduction in the time spent in ED (126.6 vs 211.3 minutes) (Dixon et al., 2009).

#### 3.9.2 Impact on emergency medical services calls

In programmes situated within EMS, there was a 26% reduction in EMS calls. In patients who had suffered a fall there was a reduction in calls and subsequent transports (Quatman‐Yates et al., 2021) and one study which looked at the impact of community paramedicine programme on patients who frequently use the service found there was a mean reduction per patient from 18 calls on average down to 8 after enrolment (Tangherlini et al., 2016). A study from Finland found that 82% of the patients assessed and treated by community paramedics did not re‐attend EMS (Hänninen et al., 2020).

#### 3.9.3 Impact on hospital admissions

Community paramedicine programmes were found to reduce rates of hospital admission. Studies reported a reduction in 30‐day readmission rates (Misra‐Hebert et al., 2021), reduced admissions (Nejtek et al., 2017) and improved quality of life. A report from Canada showed a 32% reduction in admissions to a hospital (Leyenaar, McLeod, et al., 2019) by patients receiving community paramedic intervention and in patients living in long term care receiving community paramedicine intervention they were less likely to be admitted (16.8% vs. 39.8%) (Leduc et al., 2021).

### 3.10 Economic outcomes

There were 12 studies (Agarwal et al., 2020; Ashton et al., 2017; Bennett et al., 2018; Brydges et al., 2016; Castillo et al., 2016; Dixon et al., 2009; Leyenaar, McLeod, et al., 2019; Martin‐Misener et al., 2009; Mason, O'Keeffe, et al., 2007; Thompson et al., 2014; Widiatmoko et al., 2008; Xie et al., 2021) that included some form of economic evaluation of community paramedicine programmes. Results showed that the community paramedicine programmes greatest economic impact was due to a reduction in acute healthcare utilisation through a decrease in the usual pathways of care of emergency call‐taking dispatch of paramedics, transport to the ED and ED attendance plus or minus hospital admission. See Table S2 for tabulated results.

Canadian studies showed significantly promising economic advantages of community paramedicine programmes. In a randomised controlled trial of community paramedicine programmes for low‐income seniors in subsidised housing (Agarwal et al., 2020), there was a net reduction in EMS calls, with a cost reduction of C$54–C$243 per resident in the trial, resulting in an overall cost avoided over 12 months of C$78,742 to C$355,681.

A report from Ontario, Canada (Leyenaar, McLeod, et al., 2019) demonstrated a cost avoidance of $29 million in downstream health costs in a population of 2333 patients. A net return on investment (being calculated through cost avoidance minus the cost of providing service) of C$5842 per patient per year was demonstrated, with a community paramedicine hospital discharge service creating a 50% reduction in healthcare costs and cost avoidance estimated at C$10,000 per patient. Studies originating in the USA also showed that community paramedicine programmes had an economic advantage over routine pathways of care. Through a reduction in EMS calls, ED presentations and hospital admissions one study found a return on investment of 20% in 1 year (Bennett et al., 2018). The net reduction in costs was again associated with diverting patients away from ED presentations, with one pilot study finding that “per patient, savings were US$791 for 7 days, US$3,677 for 15 days and US$538 for 30 days” (Bradley et al., 2016). Another study illustrated a 19% reduction in per‐patient costs per month in high‐risk patients.

The economic results from community paramedicine programmes were not restricted to the North American context. In the United Kingdom, paramedic practitioner programmes were found to be cost‐effective at £20,000 per Quality Adjusted Life Year (QALY) (Dixon et al., 2009). When discussing mixed models of care a programme that involved a nurse and paramedic team attending to low acuity calls found that the cost of implementation of the programme was offset by the savings from the reduction in ED presentations and hospitalisations. The programme ran over a 15‐week period and saved £29,260 (Widiatmoko et al., 2008). In Australia, a community paramedicine programme, which was implemented across 6 different sites found that should the programme sites be used consistently, annual costs saving per patient seen by the community paramedic equivalent would range from AUD$411 to AUD$998 (Thompson et al., 2014).

### 3.11 Patient health outcomes

Seven studies (Agarwal et al., 2017; Agarwal et al., 2018, 2019; Ash, 2020; Bennett et al., 2018; Hoyle S, 2012; Mason et al., 2008) provided evidence associated with whether community paramedicine programme interventions influenced patient health outcomes. See Table S3 for tabulated results. Three studies specifically reported on the change in both blood pressure and diabetes risk. In patients with hypertension community paramedic intervention showed that both systolic and diastolic blood pressure was decreased significantly (Agarwal et al., 2017; Bennett et al., 2018). Likewise, diabetes risk was decreased in 15% of participants in one Canadian study (Agarwal et al., 2017) and one US‐based study patients suffering from diabetes saw a decrease in blood glucose measurements on average of 33.7 mmol/L (Bennett et al., 2018).

When considering the quality‐of‐life measures, three studies (Agarwal et al., 2018, 2019; Ash, 2020) used this as an outcome measure. In a randomised control trial participants receiving the community paramedicine intervention showed significant results in QALYs and were more likely to be able to perform usual daily activities (odds ratio 2.6, 95% CI 1.2 to 5.8) (Agarwal et al., 2018). In a similar but separate randomised control trial of community paramedicine an increase in QALYs was also seen (mean difference 0.06, 95%CI: 0.02 to 0.10) (Agarwal et al., 2019). A US study that used EQ‐5D‐3L scores to measure the impact of a community paramedicine programme on patients found that the mean difference between pre‐ and post‐scores was statistically significant and participants scored nearly 20 points higher on the perceived quality of life (Ash, 2020).

Two studies evaluated the patient safety of community paramedicine programmes, one study found no difference in 28‐day mortality rates and a study from New Zealand showed that of only 18 cases where there was a subsequent presentation after a non‐transport decision was made, on clinical review all cases at the time the decisions were made were deemed clinically appropriate (Hoyle et al., 2012).

### 3.12 Patient satisfaction

Nine studies (Brydges, 2014; Castillo et al., 2016; Hughes & Seenan, 2021; Martin et al., 2016; Mason et al., 2008; Ruest et al., 2017; Shah et al., 2018; Swain et al., 2012; Thompson et al., 2014) either primarily investigated or reported on patient satisfaction with care provided by community paramedicine programmes. See Table S4 for tabulated results. Patients in the community paramedicine programmes found their experience to be positive (Hughes & Seenan, 2021) and were highly satisfied with the care provided (Mason et al., 2008). Patients appreciated the fact that the programmes provided the improved ability for health monitoring, provided primary healthcare needs at home which increased the sense of security as well as provided increased education on their health issues making patients feel empowered to manage their own health (Martin et al., 2016). Patient satisfaction was driven by both the professional and personal relationships that the community paramedics were able to develop throughout the community paramedicine programme model of care (Brydges, 2014).

One study investigated satisfaction differences between routine EMS models of care compared with a community paramedicine model of care (Swain et al., 2012). Both models of care were accepted by patients, but a small number of patients still did have a preference to be transported to the ED rather than having care provided in the community or at their place of residence. This was echoed in an Australian report, which also found that 2.2% of patients refused to be treated by community paramedics (Thompson et al., 2014).

### 3.13 Community paramedic satisfaction and qualitative insights into the role

Eight studies (Adio et al., 2020; Brydges et al., 2016; Clarke, 2019; Cooper et al., 2007; Harvey et al., 2021; Martin & O'Meara, 2019; O'Meara et al., 2016; Whalen et al., 2018) examined the satisfaction and qualitative experience of community paramedics (or local equivalent) operating in a community paramedicine programme. See Table S5 for tabulated results. Paramedics working in community paramedicine programmes were found to value the role and enjoyed the novel approach to care that impacted positive patient outcomes (Whalen et al., 2018) and they felt accepted to work in non‐traditional ambulance roles by other health professionals (Clarke, 2019). Paramedics felt that the key to the success of community paramedicine programmes and their role was dependent on interprofessional relationship building (Adio et al., 2020; Harvey et al., 2021; Martin et al., 2016; Schwab‐Reese et al., 2021). There were reports of opportunities to improve the experience of community paramedics such as by increasing clinically focussed graduate‐level education (Cooper et al., 2007; Martin & O'Meara, 2019), improving the communication from management staff and better communication about the role of community paramedics with other paramedic staff and allied health partners (Martin & O'Meara, 2019; Schwab‐Reese et al., 2021).

## 4 DISCUSSION

This review identified 98 studies that explored several aspects of community paramedicine, namely education, programme delivery, governance and clinical supervision, the scope of the role, and outcomes. While much of the evidence was of low quality (Burns et al., 2011), taken as a whole the results provide a comprehensive overview of the implementation of community paramedicine around the world in the last 2 years, from the inception of some of the first programmes, to modern‐day programme innovations in response to COVID‐19.

One of the core tenets of community paramedicine is the design and implementation of programmes that meet the needs of the community (CSA Group, 2017). Although the curriculum outlined by the Paramedic Foundation in the USA (Paramedic Health Solutions, 2016) provides an introductory module on the conduct of needs assessments, there is a noticeable lack of literature that critically discusses or explores the components of a community needs assessment. Given the acknowledgement that paramedicine is a profession that practises on a health‐social care continuum (Tavares et al., 2021; Williams et al., 2021) it appears timely for guidance on contemporary approaches to conducting community needs assessments that are holistic and patient‐centred, acknowledge the complex barriers to health and social care access in marginalised populations (Batt, Williams, Brydges, et al., 2021) and are co‐created with the community, instead of on their behalf. Central to delivering a service that meets the needs of the community is first educating and supporting community paramedics to do so. Despite the shift towards competency‐based education evident in the health professions over the past 10 years (Batt et al., 2020), only one study in this review (Ruest et al., 2017) identified required competencies to inform education design and delivery. Although other educational programmes may have been modelled against identified competencies, the lack of reporting on this aspect of education means there is some uncertainty in the appropriateness of education programmes, and a potential for missed opportunities in addressing community needs. It would appear prudent to first identify the competencies needed of community paramedics in specific contexts (Batt, Williams, Brydges, et al., 2021), and use these findings to design education and assessment strategies.

It is obvious that education will therefore influence the scope of the role community paramedics undertake. Evidence in this review demonstrated that the generalist pre‐registration education required by paramedics is harnessed to enable the development of community paramedics across different clinical settings and different clinical needs. This pluripotential characteristic of paramedics (Eaton, Wong, et al., 2021) enables the scope of role to reflect either the environment or community in which they work. Central to the expanded scope of role for community paramedics was the health promotion and the importance of a holistic biopsychosocial assessment. Community paramedics do not only operate in a response model, but in a proactive approach that focuses on making every contact count.

An important part of community paramedicine programme implementation is the robust evaluation used to measure its impact and ability to achieve any pre‐set goals. The most‐reported outcome measures found in the literature was the impact of community paramedicine programmes on rates of transport by paramedics and rates of ED presentations. There are limitations in using this as a sole outcome measure to evaluate effectiveness (Uscher‐Pines et al., 2013). It has been highlighted that community paramedicine programmes often aim to serve the most vulnerable and complex patients who may require intense healthcare utilisation (Dainty et al., 2018). This is particularly the case in programmes that care for previously identified frequent attenders which have been noted in the literature to not engage in preventative healthcare measures (Hudon et al., 2016). There is also a natural decrease in ED presentations in some patient groups over time and this may bias results in evaluations solely looking at rates of transport and ED presentations (Hudon et al., 2016). For this reason, the outcomes measure of rates of transportation and presentation to EDs should be one part of a robust evaluation when looking at the impact of community paramedicine programmes. Finally, the patient outcomes measured were heterogeneous with many patient outcomes measures showing a positive impact. Quality of life measures appear to show the positive impact of community paramedicine and would be one of the more appropriate outcome measures to be used when considering patient‐level outcomes (Nolan et al., 2018).

### 4.1 Limitations

Although the restricted review methodology used could be questioned, we aimed to address this via a robust auditing process and full text screening review. There is a possibility that literature not available in English may have added value to be included in this review and thus results pertaining to the review aims may have been missed. Despite this, literature from countries where English is not the primary language were still able to be included through finding English translations and were included where possible.

### 4.2 Implications for future research

The significant amount of literature included highlights the major areas of evidence supporting community paramedicine implementation as well as identifying gaps. Knowledge gaps found include a lack of literature on the conduct of community needs assessments, education requirements, as well as core curriculum needs for community paramedicine. These gaps were identified despite a thorough grey literature search and should be areas of focus for further research pertaining to community paramedicine.

### 4.3 Recommendations

This review found that community paramedicine programmes are adaptive to community needs. It is important for stakeholders implementing community paramedicine programmes to identify community needs early. This will ensure that community paramedicine programmes are fit for purpose and adaptive as community needs change over time. Education required for community paramedics, and the scope of the role they undertake, differs across continents, and across different jurisdictions within each country. Therefore, educational programmes should be structured to support the development of predetermined competencies. There was a lack of standardisation in the implementation of governance and supervision models to support community paramedics in both their role and development and this should be addressed in future developments. Finally, the inconsistency in outcomes reported in the literature demonstrate a gap in the current evidence. It is therefore important to consider quantitative, qualitative and economic analysis designs when looking to evaluate the impact of community paramedicine programmes.

## 5 CONCLUSION

This review identified and explored community paramedicine literature focused on five key areas: education, models of delivery, governance and clinical support, the scope of the role and outcomes associated with community paramedic models. The findings of this review demonstrate a lack of research and understanding of the education and scope of the role of community paramedics, and also highlighted a need to develop common approaches to education and scope while maintaining flexibility in addressing community needs. There was a lack of standardisation in the implementation of governance and supervision models which may prevent community paramedicine from realising its full potential. Finally, although there has been an increased focus on outcomes in the literature, such reporting is inconsistent. This inconsistency, and the gaps evident across the other areas of focus, makes it difficult to articulate what community paramedicine programmes can achieve and their impacts on the healthcare system.

## AUTHOR CONTRIBUTION

BS, GE and AB conceived the study. BS, GE, AB, K‐AB and BW equally designed the study approach. BS, GE, AB, CL, MN, ML, GW and JH undertook the review and data collection and data analysis. BS, GE, CL, AB, ML and MN interpreted the data. BS, GE, AB, BW and K‐AB drafted the manuscript and circulated to authors for contribution. All authors edited drafts and approved the current manuscript for publication. BS is the author responsible for the overall content as the guarantor.

## FUNDING INFORMATION

This study was commissioned and funded by the Pre‐Hospital Emergency Care Council and was awarded following a competitive tendering process.

## CONFLICT OF INTEREST

No conflict of interest has been declared by the author(s).

## Supporting information


Table S1

## Data Availability

Data sharing is not applicable to this article as no new data were created or analysed in this study.
