# Supplementary material for: The development of community paramedicine; a restricted review
Source: Health Soc Care Community. 2022 Sep 5;30(6):e3547–61. doi: 10.1111/hsc.13985 (PMC10087318; doi:10.1111/hsc.13985)
Supplement: Supplementary file 1 — Table S1 [file HSC-30-e3547-s001.zip › hsc13985-sup-0001-Tables.docx]

# Supplementary Material

*Table 1. Impact on emergency calls, rates of transportation and hospital admissions*

| **Title** | **Author** | **Year** | **Origin** | **Aim of study** | **Emergency calls, Transportation and Admission Outcomes** |
| --- | --- | --- | --- | --- | --- |
| Reducing 9-1-1 Emergency Medical Service Calls By Implementing A Community Paramedicine Program For Vulnerable Older Adults In Public Housing In Canada: A Multi-Site Cluster Randomized Controlled Trial. | Agarwal | 2019 | Canada | Evaluate the change in mean EMS calls at the building-level, comparing intervention and control buildings, across multiple community sites. | Intention-to-treat analysis showed no significant difference in EMS calls (mean difference 0.37/100 apartment units/month, 95%CI:0.98 to 0.24). Sensitivity analysis excluding data from 2 building pairs with eligibility changes after intervention initiation revealed a significant difference in EMS calls in favour of the intervention buildings (mean difference 0.90/100 apartment units/month, 95%CI:1.54 to0.26). |
| Community Paramedicine Applied in a Rural Community | Bennett | 2020 | United States | The objective of this study was to determine if the CP program reduced ED visits in Abbeville while improving patient outcomes | ED visits among participants decreased by 58.7%, substantially different from the 4% increase in the comparison group (P<.0001). Similarly, inpatient admissions decreased by 68.8% compared to an increase in admissions of 187.5% among the comparison group (P=.045). Length of stay decreased by 15.7% for participants yet increased by 162.5% among the comparison group (P=.03). Among the CP program participants with hospitalisation, there was a 41.2% reduction in 30-day readmissions, compared to a 35.9% increase among the comparison group; this reduction was even higher among those with COPD (75% decrease). It should be noted that the 30-day readmissions rate was significantly impacted by 1 participant, who accounted for 16 visits; without that participant's data, there was an 83.1% decrease in 30-day readmissions, significantly higher than the comparison group (P<.0001). |
| The business case for community paramedicine: lessons from Commonwealth Care Alliance Pilot Program | Bradley | 2016 | United States | Summarises acute community care program business case | In the first year of operations, 126 patients received an ACC visit. Out of those patients, 81 percent remained home under the care of ACC paramedics while 19 percent were transported for emergent care needs. |
| Mobile Integrated Healthcare: Preliminary Experience and Impact Analysis with a Medicare Advantage Population | Castillo | 2016 | United States | Aims to describe and analyse the initial experience and preliminary impact of an MIH intervention delivered at scale for a high-risk subpopulation. | 21% decrease in emergency department utilisation, 37% decrease in inpatient PMPM cost, 40% decrease inpatient utilisation, all measures reached statistical significance. |
| Mobile Integrated Health Care and Community Paramedicine: An Emerging Emergency Medical Services Concept. | Choi | 2016 | United States | Literature review of MIH CP | Reduction in ED transports and hospital admissions. Not conclusive on impact |
| The emerging role of the emergency care practitioner | Cooper | 2004 | UK | To examine the emerging role of the emergency care practitioner (ECP) with comparisons to paramedic practice. | ECPs were more likely to treat patients on scene than paramedics (p = 0.007); 28% (48 of 170) by ECPs compared with 18% (59 of 331) by paramedics. None of the ECPs or paramedics patients was subsequently conveyed within 24 hours. Patients were conveyed, either by A&E, urgent transfer, patient transfer, rapid response, air ambulance, or ECP vehicles. ECPs arranged conveyance for 50% (85 of 170) of their patients while paramedics conveyed 64% (212 of 331) (p = 0.000) |
| Is it cost effective to introduce paramedic practitioners for older people to the ambulance service? Results of a cluster randomised controlled trial | Dixon | 2008 | UK | To assess the cost-effectiveness of the paramedic practitioner (PP) scheme compared with usual emergency care. | Whereas the intervention group received more PP contact time, it reduced the proportion of emergency department (ED) attendances (53.3% vs 84.0%) and time in the ED (126.6 vs 211.3 minutes). There was also some evidence of increased use of health services in the days following the incident for patients in the intervention group. Overall, total costs in the intervention group were £140 lower when routine data were considered (p = 0.63). When the costs and QALY were considered simultaneously, PP had a greater than 95% chance of being cost-effective at £20 000 per QALY. |
| The effect of a mobile integrated health program on health care cost and utilization. | Gingold | 2021 | United States | To measure the effect of a mobile integrated health community paramedicine (MIH-CP) transitional care program on hospital utilisation, emergency department visits, and charges. | No significant difference in short-term health care utilisation or charges between patients enrolled in an MIH-CP transitional care program and propensity-matched controls. |
| Patients' experiences of care provided by emergency care practitioners and traditional ambulance practitioners: A survey from the London Ambulance Service | Halter | 2006 | UK | To compare patients’ experiences of ECP care with that from traditional ambulance practitioners (state-registered paramedic or emergency medical technician). | 80.2% of those attended by SRPara or EMTs were conveyed to the emergency department compared with 58.0% of those attended by ECPs (x2= 52.08, df = 1, p,0.001 |
| Patients Seeking Retreatment after Community Paramedic Assessment and Treatment: Piloting a Community Paramedic Unit Program in Southwest Finland | Hanninen | 2020 | Finland | To categorise CP unit patients seeking retreatment after a CP unit visit and investigate links between CP unit actions and patients seeking retreatment. | The main results show that 82% of the patients assessed and treated by the CP unit did not re-attend |
| Impact of a Novel Collaborative Long-Term Care EMS Model: A Before-and-After Cohort Analysis of an Extended Care Paramedic Program | Jensen | 2016 | Canada | The objective of this study was to measure differences in the delivery of emergency care for LTC residents with acute illnesses or injuries attended by ECP or emergency paramedics, measured primarily with a number of transports to the ED, as well as EMS response and scene time, patient ED length of stay, EMS time in the ED, hospital admission, and relapse back to EMS after calls ending in no transport. | Reduced number of transports, improved measures of time periods. |
| The Safety and Effectiveness of On-Site Paramedic and Allied Health Treatment Interventions Targeting the Reduction of Emergency Department Visits by Long-Term Care Patients: Systematic Review. | Leduc | 2020 | International | A systematic review of the literature to determine, among long-term care patients, what is the effectiveness and safety of interventions that evaluate and treat patients on-site, avoiding unscheduled transport to the ED. | Two studies, representing one program, utilised extended care paramedics who responded to calls for acute issues in long-term care centres, such as abdominal pain, diabetic problems and traumatic injuries. They found a 29.3% decrease in overall ED transfers after the program was implemented (p<.001) and that patients treated by an extended care paramedic were less likely to visit the ED than those treated by an emergency paramedic (45.3% vs 92.7%, p<.001). The second paper found patients seen by the extended care paramedic were less likely to be admitted (16.8%) compared to those seen by an emergency paramedic (39.8%) |
| Report on the status of community paramedicine in Ontario | Leyenaar | 2019 | Canada | Report summarising community paramedicine in Ontario | The programme overall results showed that for patients enrolled in the program there was a 26% reduction in 9-1-1 calls, a 26% reduction in emergency department visits, a 32% reduction in hospital admissions, and a 41%reduction in hospital readmissions. Findings from Toronto published over the past year have shown that Assessment and Referral Programmes improved access to home care services by 24%, led to an average increase of 17.4 hours in total home care services per person while reducing 9-1-1 calls by 10% and ambulance transports to emergency departments by 7% over the study period |
| Effectiveness of emergency care practitioners working within existing emergency service models of care | Mason | 2007 | UK | (1) To evaluate appropriateness, satisfaction and cost of ECPs compared with the usual service available in the same healthcare setting, (2) to increase understanding of what effect, if any, ECPs are having on the delivery of health services locally and (3) to evaluate whether ECP working yields cost savings. | CPs carried out fewer investigations, provided more treatments and were more likely to discharge patients home than the usual providers. |
| Safety of paramedics with extended skills | Mason | 2008 | United States | The objectives were to evaluate the safety of clinical decisions made by Paramedic Practitioners operating within the new service. | Overall, patients in the intervention group were less likely to attend the ED (relative risk [RR] 0.72, 95% CI = 0.68 to 0.75) or require hospital admission within 28 days (RR 0.87, 95% CI = 0.81 to 0.94) and experienced a shorter total episode time (235.07 min vs. 277.8 min, 95% CI of difference) 59.5 to -25.0). There was no statistically significant difference in 28-day mortality (RR 0.87, 95% CI = 0.63 to 1.21). |
| Healthcare utilization and patient and provider experience with a home visit program for patients discharged from the hospital at high risk for readmission. | Misra-Hebert | 2021 | United States | The goals of this study were to assess the association with health care utilisation and mortality for patients at high risk for readmission who participated in the post-discharge home visit program and to examine provider and patient experience regarding program participation. | Findings supported a lower 30-day readmission association within the intervention groups. |
| A pilot mobile integrated healthcare program for frequent utilizers of emergency department services | Nejtek | 2017 | United States | To examine whether or not a mobile integrated health (MIH) program may improve health-related quality of life while reducing emergency department (ED) transports, ED admissions, and inpatient hospital admissions in frequent utilisers of ED services | The results from this small retrospective program evaluation suggest that MIH participation was associated with improved quality of life, fewer ED transports, fewer ED admissions, and reduced inpatient admissions |
| Assessment of Fall-Related Emergency Medical Service Calls and Transports After a Community-Level Fall-Prevention Initiative | Quatman-Yates | 2021 | United States | To study the impact of a community paramedic programmes optimization of a fall prevention system entailing a clinical pathway and learning health system (called Community-FIT) on community-level fall-related emergency medical service utilisation rates | The community paramedicine program demonstrated a reduction in fall-related calls and transports. |
| Evaluating the impact on 911 calls by an in-home programme with a multidisciplinary team | Ruest | 2012 | Canada | Review whether the use of community paramedics as part of an integrated health care tams can reduce 911 calls | 129 patient interactions by community paramedics only 15 incidents resulted in ED visits using 911 |
| The HOME Team: Evaluating the Effect of an EMS-based Outreach Team to Decrease the Frequency of 911 Use Among High Utilizers of EMS. | Tangherlini | 2016 | United States | examined the effectiveness to reduce repeat use of EMS. | The HOME Team undertook 320 distinct contacts of 59 frequent users during the study period, with an average of 5.42 contacts per patient. The maximum number of contacts was 46. The average use of EMS services by the identified frequent users before first contact was 18.72 (SD=19.40). The average use after first contact was 8.61 (SD=10.84). The mean difference was 10.11; 95% CI, 4.36-15.86; P-value <.001 |
| HWA Expanded Scopes of Practice program evaluation: Extending the Role of Paramedics sub-project | Thompson | 2014 | Australia | Report on the Extended Care Paramedic program in originally developed in South Australia Ambulance Service and implemented within 5 sites in New South Wales | Overall, 62% of eligible patients were treated at a private residence (ranging from 50% at one site to 77% at another site). A high proportion of patients (72.5%, range 65% to 78% at different sites) seen by ECPs did not require transport to hospital. |

*Table 2. Economic outcomes results*

| **Title** | **Author** | **Year** | **Origin** | **Aim of study** | **Economic Outcomes** |
| --- | --- | --- | --- | --- | --- |
| Cost-effectiveness analysis of a community paramedicine programme for low-income seniors living in subsidised housing: the community paramedicine at clinic programme (CP@clinic). | Agarwal | 2020 | Canada | To evaluate the cost-effectiveness of the CP@clinic programme compared with usual care for low-income seniors living in subsidised (social) housing | The cost of running the intervention in all five RCT sites for one year was $128462. Due to the reduction of 157.8 EMS calls over the intervention year, the estimated cost avoided during the RCT ranged from $78742 to $355681. This resulted in a cost offset of $54- $243 per resident |
| Conserving Quality of Life through Community Paramedics. | Ashton | 2017 | Canada | To determine whether community paramedicine services (the intervention through home visits) would have a positive economic impact through influencing the self-perceived quality of life and determining a monetised value | The economic impact of CP through conserving the quality of life was monetised through conversion to QALYs and consideration of the cost of the intervention. Per client marginal costs for one year’s CP service through this study was calculated to be $5,675 for Renfrew and $5,731 for Hastings. On that basis, the cost to realise a QALY through this community paramedicine intervention was $67,560 for Renfrew and $76,413 for Hastings. |
| Community Paramedicine Applied in a Rural Community. | Bennett | 2017 | United States | The aim was to determine if the CP program reduced ED visits in Abbeville while improving patient outcomes | The Abbeville CP program estimated the cost per visit to be $205.78. This is based upon $4,101.93 in start-up costs; $8,473.20 for equipment purchases; $73,127.56 in personnel costs; and $5,251.55 in travel and maintenance costs, for a total of $90,954.24 for the year of 2015. Since the CP program is part of the existing EMS infrastructure, additional costs for the start-up of the CP program were minimised. Using data supplied by the AAMC cost report, the estimated cost of an average inpatient day was estimated to be $1,531, an ED visit to be $449, and an EMS call to be $312. Given the annualised reduction in ED visits (124), inpatient days (28), and EMS calls (34), a positive marginal benefit to the local health care system was estimated to be at least $18,198, or a return on investment of more than 20%. |
| The business case for community paramedicine: lessons from Commonwealth Care Alliance Pilot Program | Bradley | 2016 | United States | Summarises acute community care program business case | Under the pilot program, patients diverted from the ED had lower average costs than those not diverted on a patient-episode basis (per patient savings were $791 for seven days, $3,677 for 15 days, and $538 for 30 days). Accounting for service costs, utilisation, and ED diversion rates, as well as anticipated ACC operating costs and expected ACC patient volume in different geographic regions of Massachusetts, the analysis suggests substantial savings potential |
| Mobile Integrated Healthcare: Preliminary Experience and Impact Analysis with a Medicare Advantage Population | Castillo | 2016 | United States | Aims to describe and analyse the initial experience and preliminary impact of an MIH intervention delivered at scale for a high-risk subpopulation. | 19% decrease in emergency department per member per month (PMPM) cost |
| Is it cost effective to introduce paramedic practitioners for older people to the ambulance service? Results of a cluster randomised controlled trial | Dixon | 2008 | UK | To assess the cost-effectiveness of the paramedic practitioner (PP) scheme compared with usual emergency care. | Overall, total costs in the intervention group were £140 lower when routine data were considered (p = 0.63). When the costs and QALY were considered simultaneously, PP had a greater than 95% chance of being cost-effective at £20 000 per QALY. |
| Report on the status of community paramedicine in Ontario | Leyenaar | 2019 | Canada | Report summarising community paramedicine in Ontario | Cost avoidance of over $29M in downstream health system costs was achieved amongst the 2,333 patients that participated in this program during the evaluation period. The overall reductions found in health system utilisation generated an estimated $7,279 in cost avoidance for the healthcare system per patient per year, with the cost of community paramedic and equipment of $1,455 per patient per year, a net return on investment (cost avoidance - the cost of providing service) $5,842 per patient per year. A community paramedicine enabled hospital discharge program from Sudbury resulted in a 50% reduction in total health care costs per patient and estimated cost avoidance to be $10,000 per patient enrolled. |
| Cost effectiveness and outcomes of a nurse practitioner paramedic family physician model of care: the Long and Brier Islands study | Martin-Misener R | 2009 | Canada | Do patients in a collaborative model demonstrate evidence of improved psychosocial adjustment and less expenditure of health care resources over time | The NP paramedic physician model of primary health care services increases access to health care services and is a cost-effective model of health care for rural communities with low emergency call volumes |
| Effectiveness of emergency care practitioners working within existing emergency service models of care | Mason | 2007 | UK | (1) To evaluate appropriateness, satisfaction and cost of ECPs compared with the usual service available in the same healthcare setting, (2) to increase understanding of what effect, if any, ECPs are having on the delivery of health services locally and (3) to evaluate whether ECP working yields cost savings. | CPs carried out fewer investigations, provided more treatments and were more likely to discharge patients home than the usual providers. Patients were satisfied with the care received from CPs, and this was consistent across the three different settings. It was found that CPs are working in different settings across traditional professional boundaries and are having an impact on reconfiguring how those services are delivered locally. Costs information (based on deployment within ambulance service) indicated that CP care may be cost effective in that model of CP working. |
| HWA Expanded Scopes of Practice program evaluation: Extending the Role of Paramedics sub-project | Thompson | 2014 | Australia | Report on the Extended Care Paramedic program in originally developed in South Australia Ambulance Service and implemented within 5 sites in New South Wales | Scenario analysis shows that if all implementation sites saw six ECP patients each shift (that is, six days for each site for 365 days per year) and the same levels of ED avoidance rates seen during implementation were maintained all sites would be highly cost-effective with annual cost savings ranging from $411 per patient at ERP5 to $998 at ERP2. |
| Developing a new response to non-urgent emergency calls: evaluation of a nurse and paramedic partnership intervention | Widiat-moko | 2008 | UK | To investigate the cost-effectiveness of a new service development whereby a nurse and a paramedic working in partnership attended non-urgent emergency calls | Conservative estimate of the costs incurred in the A&E department and the subsequent hospitalisation rate; the modelling demonstrated that the project saved £29 260 during its 15 weeks. The cost of providing the pilot service was compensated by the savings made from the reduced use of the A&E department and subsequent hospitalisation |
| Economic Analysis of Mobile Integrated Health Care Delivered by Emergency Medical Services Paramedic Teams. | Xie F | 2021 | Canada | "To compare time on task and cost between MIH and ambulance delivered by NEMS from a public payers perspective" | Community paramedicine "was associated with reduced ED transport and saved substantial savings of EMS staff time and resources compared with an ambulance for the matched emergency calls." |

*Table 3. Patient health outcomes results*

| **Title** | **Author** | **Year** | **Origin** | **Aim of study** | **Patient Outcomes** |
| --- | --- | --- | --- | --- | --- |
| Effectiveness of a community paramedic-led health assessment and education initiative in a seniors' residence building: the Community Health Assessment Program through Emergency Medical Services (CHAP-EMS). | Agarwal | 2017 | Canada | The aim was to evaluate whether a weekly 8-hour CHAP-EMS program was associated with changes in (1) number of emergency EMS calls (9-1-1) from the seniors’ residence building, (2) mean blood pressure (BP) of participants and (3) diabetes risk profile of participants after one year of implementation. Intervention: CHAP-EMS is a low-cost, community paramedicine program designed to assess community-dwelling seniors for lifestyle risk factors that may impact their health and wellbeing and to provide targeted education to address the pertinent risk factors | At baseline, 42% of participants had elevated blood pressure. Systolic blood pressure decreased significantly by the participant's 3rd visit to CHAP-EMS and diastolic by the 5^th^ visit (p< .05). At baseline, 19% of participants had diabetes; 67% of those undiagnosed had a moderate or high risk based on the Canadian Diabetes Risk (CANRISK) assessment. 15% of participants dropped one CANRISK category (e.g., high to moderate) during the intervention. |
| Evaluation of a community paramedicine health promotion and lifestyle risk assessment program for older adults who live in social housing: a cluster randomized trial. | Agarwal | 2018 | Canada | The aim was to use a randomized controlled trial (RCT) to determine if implementing CP@clinic decreases mean ambulance calls (primary outcome) in the intervention versus control buildings, measured at the building level. Secondary outcomes were improvement in risk-factor profiles and HRQoL among older adults living in subsidised community housing (individual-level measures and analysis) | Residents living in the intervention buildings showed significant improvement compared with those living in control buildings in quality-adjusted life years (QALYs) (mean difference 0.09, 95% CI 0.01 to 0.17) and ability to perform usual activities (odds ratio 2.6, 95% CI 1.2 to 5.8). Those who received the intervention had a significant decrease in systolic (mean change 5.0, 95% CI 1.0 to 9.0) and diastolic (mean change 4.8, 95% CI 1.9 to 7.6) blood pressure. |
| Reducing 9-1-1 Emergency Medical Service Calls By Implementing A Community Paramedicine Program For Vulnerable Older Adults In Public Housing In Canada: A Multi-Site Cluster Randomized Controlled Trial. | Agarwal | 2019 | Canada | Evaluate the change in mean EMS calls at the building-level, comparing intervention and control buildings, across multiple community sites. Intervention: Community paramedic led risk assessment, disease prevention and health promotion sessions weekly in common areas of intervention buildings | At the individual level, there was a significant QALY increase (mean difference 0.06, 95%CI: 0.02 to0.10) and blood pressure decrease (systolic mean change 3.65 mmHg, 95%CI: 2.37 to 4.94; diastolic mean change 2.03 mmHg, 95%CI: 1.00 to 3.06). |
| Quality of Life for Persons with Chronic Disease Utilizing Mobile Integrated Healthcare | Ash | 2020 | United States | The study aimed to analyse the relationship between NCD type; age; gender; duration of participation in MIH-CP; hospital readmission; and self-reported, perceived QOL as measured by the EQ-5D-3L for those who received services from the MIH-CP program | No variables were found to be related at statistically significant levels to self-reported, perceived QOL as measured by EQ-5D-3L after completing the MIH program 3. The difference in scores between the pre-test and post-test administration of the EQ-5D-3L was statistically significant (p = .000). The mean difference in the scores was 19.88, which means that the perceived QOL after the intervention increased by nearly 20 points. This difference may indicate an increase in perceived QOL upon graduation from the MIH-CP program |
| Community Paramedicine Applied in a Rural Community. | Bennett | 2017 | United States | The aim was to determine if the CP program reduced ED visits in Abbeville while improving patient outcomes | Hypertensive patients decreased an average of 7.2 mmHg (P<.0001) in systolic blood pressure and 4.0 mmHg (p<.0001) in diastolic blood pressure. Diabetic patients decreased blood glucose by an average of 33.7mmol/L (p=.0013). |
| Introduction of an extended care paramedic model in New Zealand | Hoyle | 2012 | New Zealand | The study aimed to determine the rate of treatment in the community and to examine any acute hospital presentation within 7 days from ECP presentation. | These 18 cases were reviewed by an emergency medicine specialist (AHS) and in each case, the initial ECP management was considered to be appropriate at the time. The clinical presentation in the ED was either the result of deterioration of the clinical condition despite an appropriate management plan (four cases) or a presentation to the ED that was not warranted based on the severity of the clinical condition (14 cases). |
| Safety of paramedics with extended skills | Mason | 2008 | United States | The objectives were to evaluate the safety of clinical decisions made by Paramedic Practitioners operating within the new service. | There was no statistically significant difference in 28-day mortality (RR 0.87, 95% CI = 0.63 to 1.21). |

*Table 4. Patient satisfaction results*

| **Title** | **Author** | **Year** | **Origin** | **Aim of study** | **Outcome result notes** |
| --- | --- | --- | --- | --- | --- |
| A Case Study of Older Adult Experiences with a Novel Community Paramedicine Program | Brydges | 2014 | Canada | Aim to understand older adults' experiences with a novel community paramedicine program, the Cardiovascular Health Awareness Program by EMS (CHAP-EMS), operating in a subsidised housing building in Hamilton. | The perceptions and experiences of older adults residing in a building with a community paramedicine program are complex, however, it was clear that distinct themes emerged from these views and experiences. The participants of the program highly valued the program, the paramedics, and the change in social dynamics to the building. The program offered an opportunity for social participation, an increase in access to support and resources, and a trusting avenue to discuss their health. Further, the paramedics provided support, relationships (both professional and personal), a sense of community, and opportunities for participation and engagement amongst building residents |
| Mobile Integrated Healthcare: Preliminary Experience and Impact Analysis with a Medicare Advantage Population | Castillo | 2016 | United States | Aims to describe and analyse the initial experience and preliminary impact of an MIH intervention delivered at scale for a high-risk subpopulation. | Member experience satisfaction scores and patient activation measures also showed favourable preliminary trends |
| Community paramedicine home visits: patient perceptions and experiences | Hughes | 2021 | UK | To explore patient perceptions and experiences of CP home visits delivered by specialist paramedics (SPs) in a Scottish urban general practice home-visit setting. | Patient perceptions and experience of CP were positive, with patients accepting this model of care. Opportunities to improve healthcare, including better continuity of care and health monitoring found |
| Consumer perspectives of a community paramedicine program in rural Ontario. | Martin | 2016 | Canada | The aim was to report on a community paramedicine program in rural Ontario, Canada, through the perceptions and experiences of consumers | Three main interlinked themes were identified: (i)improved health monitoring and primary health care access close to home; (ii) improved sense of security and support for vulnerable residents in the community; and(iii) improved education and empowerment for better health management. |
| Safety of paramedics with extended skills | Mason | 2008 | United States | The objectives were to evaluate the safety of clinical decisions made by Paramedic Practitioners operating within the new service. | Patients in the intervention group were more likely to report being highly satisfied with their health care episode (RR 1.16, 95% CI = 1.09 to 1.23). |
| Community health evaluations completed using paramedic services (CHECUPS): design and implementation of a new community based health program | Ruest | 2017 | Canada | Overview of design and evaluation of the implementation of CHECUPS program | Approximately 82% felt that their overall health improved, and 99% felt that their understanding of their medical conditions allowed them to better manage their conditions. All patients treated by community paramedics stated that they were either "very satisfied" or "satisfied" with the care provided by those community paramedics |
| Improving the ED-to-Home Transition: The Community Paramedic-Delivered Care Transitions Intervention-Preliminary Findings. | Shah | 2018 | United States | To describe an innovative approach to improve the ED-to-home transition. | Participants reported the CTI Program as highly acceptable, with patient and caregiver participants reporting being likely or extremely likely in the future to choose an ED with the CTI Program over one without the program (244 (76.2%); 69 (83.1%) respectively |
| Patient satisfaction and outcome using emergency care practitioners in New Zealand. | Swain | 2012 | New Zealand | To determine whether patients found the UCC model of service both acceptable and effective, and to ascertain whether there was any difference in satisfaction with the care provided by the two groups of paramedics, EAS or ECP | From questions put to all patients, it was determined that both paramedic groups arrived promptly and that their clinical assessments were deemed to be appropriate. Satisfaction with the care provided was rated very highly (greater than 9/10). However, ECP assessments and treatment took on average 20 min longer than those of EAS crews (P<0.0001). Five of the 38 ECP patients treated at home would have preferred to be taken to hospital, but the ECPs determined that hospital admission was not required. |
| HWA Expanded Scopes of Practice program evaluation: Extending the Role of Paramedics sub-project | Thompson | 2014 | Australia | Report on the Extended Care Paramedic program in originally developed in South Australia Ambulance Service and implemented within 5 sites in New South Wales | Evidence from the patient survey confirmed that there was a very high level of consumer satisfaction with the ERP model at all sites. In general, patients reported that the ECP listened and communicated well, examined them thoroughly, provided effective treatment and seemed comfortable dealing with their problems. A small group of patients would have preferred more information regarding recovery and self-care, suggesting a target area for future improvements. Satisfaction ratings were very high. Respondents were highly satisfied with waiting times, the care they received, and their overall experience of the ambulance services involved in the trial. Clear communication and information provision were the main factors that predicted overall satisfaction. Overall, 49 consumers refused treatment by an ECP, representing 2.2% of cases. |

*Table 5. Community paramedic satisfaction and qualitative insights results*

| **Title** | **Author** | **Year** | **Origin** | **Aim of study** | **Qualitative outcomes** |
| --- | --- | --- | --- | --- | --- |
| Community Paramedics' Perception of Frequent ED Users and the Community Paramedicine Program: A Mixed-Methods Study. | Aiyedun | 2020 | United States | Addresses the following research questions: 1. What are the views of paramedics towards frequent emergency department users and underlying causes? 2. What are the views of paramedics about the community paramedicine program related to its relevance and administration, and their personal experiences and professional competencies? | Community paramedicine provides an opportunity to help underserved frequent attendance populations. With specific training, protocols and screening, goal setting CP are well suited to provide improved care for patients with frequent attendance. Rotation model is not viewed favourably as more than 30 days is needed to make an impact. Programmes need interdisciplinary teams to service frequent attendance |
| The CHAP-EMS health promotion program: a qualitative study on participants' views of the role of paramedics. | Brydges | 2016 | Canada | This study sought to understand participants perceptions of paramedic providers to explore their role in this unique practice setting and ultimately create an emerging framework to examine paramedic roles in community paramedicine programmes. | This study found that paramedics had dual roles as advocates for health and wellbeing and as experts in providing emergency care. Results from this study have informed an emerging framework for understanding paramedic roles in community paramedicine settings in multiple contexts: paramedics as trusting health care professionals; paramedics as patient advocates; and lastly, paramedics as emergency experts |
| What are the clinical practice experiences of specialist and advanced paramedics working in emergency department roles? A qualitative study. | Clarke | 2019 | UK | This study aimed to explore the lived experiences of paramedics who have made the transition from the ambulance service to specialist/advanced ED roles in the United Kingdom, and to explore how working in this new clinical environment influenced their clinical practice | While role transition to the ED represents a turbulent period for paramedics, elements of paramedic practice transfer directly into new roles and contribute to effective practice. The paramedics in this study found that they were accepted and supported to work in the ED setting and spoke positively of expanding their roles into other areas of the ED in the future. A significant barrier to current clinical practice emerges from a lack of access to medicines which impacts directly on the patient experience. The change in legislation to allow independent prescribing for advanced paramedics must be supported by ED managers, and interim improvements are required to extend existing PGDs to include paramedics; ultimately this will improve the quality and safety of the care they can provide, as well as the patient experience |
| Collaborative practices in unscheduled emergency care: role and impact of the emergency care practitioner--qualitative and summative findings. | Cooper | 2007 | UK | Through semi-structured interviews focussed on the ECP role and collaborative experiences. | Observational records and interviews showed that ECPs numerous links with other professions were influenced by three major themes as follows. (i) The ECP role: for example, restricted transport codes of communication, focus on reducing admissions, frustrations about patient tasking and conflicting views about leadership and teamwork. (ii) Education and training: drivers for multi-professional clinically focussed graduate-level education, requirements for skill development in minor injury units (MIUs) and general practice, and the need for clinical supervision/mentorship. (iii) Cultural perspectives: a crew room blue-collar view of inter-professional working versus emerging professional white-collar views, power and communication conflicts, and a lack of understanding of the ECPs role. |
| The ambulance service advanced practitioner's role in supporting care homes: a qualitative study of care staff experiences | Harvey | 2021 | UK | Investigated the experiences and needs of the care home staff who use the ambulance service, advanced practitioner model. | The most frequently stated benefit of the advanced practitioner service was that it enabled care home residents to be managed within the community and avoid hospital attendance or admission. Care homes are issued with guidance to support decisions about the healthcare of their residents, but most participants preferred to exercise their own judgement in making these choices. While some comments were made regarding the negative attitude of some advanced practitioners responding to the telephone referrals, overall, the positive and constructive relationships between the service and care homes were considered to be of high value. It should also be noted that the care homes that referred residents to the advanced practitioner service less often were more likely to highlight the positive relationships with their GPs and community nursing teams. |
| Perspectives from the frontline of two North American community paramedicine programmes: an observational, ethnographic study. | Martin | 2019 | Canada | The purpose of this study was to identify the motivations, job satisfaction and challenges of community paramedics | Transitional challenges facing community paramedics include navigating untraditional roles, managing role boundary barriers and a lack of self-regulation. This study highlighted that scepticism, criticism and misunderstanding caused anxiety for participants transitioning into community paramedic roles, highlighting that improved education and communication from paramedic service management with internal staff and allied health partners might improve this transitional process. |
| Community paramedicine model of care: an observational, ethnographic case study. | O’Meara | 2016 | Canada | The study aimed to identify and analyse how community paramedics create and maintain new role boundaries and identities in terms of flexibility and permeability and through this develop and frame a coherent community paramedicine model of care that distinguishes the model from other innovations in paramedic service delivery | Community engagement and situated practice distinguish community paramedicine models of care from other paramedicine and out-of-hospital health care models. Successful community paramedicine programmes are integrated with health, aged care and social services and benefit from strong governance and paramedic leadership. |
| The novel role of paramedics in collaborative emergency centres aligns with their professional identity: A qualitative analysis. | Whalen | 2018 | Canada | Ascertain the attitudes, feelings and experiences of paramedics working within the Nova Scotia Collaborative Emergency Centre construct within an interdisciplinary team | Four dominant themes were identified: 1) inter-professional relationships; 2) leadership support; 3) value to the community, and 4) paramedic identity. Paramedics enjoy working in this novel role and believe it aligns with their professional identity. High levels of patient and community satisfaction were reported |
