# Supplementary material for: The development of community paramedicine; a restricted review
Source: Health Soc Care Community. 2022 Sep 5;30(6):e3547–61. doi: 10.1111/hsc.13985 (PMC10087318; doi:10.1111/hsc.13985)
Supplement: Supplementary file 1 — Table S1 [file HSC-30-e3547-s001.zip › hsc13985-sup-0002-Appendixes.docx]

# Appendix A. Search strategy

Medline (Ovidsp) example- (search strategy was adapted to suit idiosyncrasies of each database):

1. Allied Health Personnel/ and emergenc*.mp.

2. Emergency Medical Technicians/

3. (paramedic* or ((emergency or ambulance) adj3 (technician? or practitioner? or staff* or personnel or workforce))).tw.

4. 1 or 2 or 3

5. exp General Practice/

6. general practitioners/ or physicians, family/ or physicians, primary care/

7. Primary Health Care/

8. Community Medicine/ or Community Health Services/ or Rural Health Services/

9. After-Hours Care/

10. Ambulatory Care Facilities/

11. Office Visits/

12. ((family or general) adj3 (practi* or doctor? or physician?)).tw.

13. (primary adj (care or healthcare or "health care")).tw.

14. (community adj2 (care or medicine or service?)).tw.

15. ("out of hours" or ooh or walk in or walk-in).tw.

16. ((health* or medical or ambulatory) adj2 (centre? or center? or clinic?)).tw.

17. *Triage/

18. triage.ti.

19. (Remote Consultation/ or Triage/) and Telephone/

20. exp Call Centers/

21. (helpline? or help line? or hotline? or hot line? or call centre? or call center?).tw.

22. (telephone? adj3 (service? or centre? or center? or triage)).tw.

23. ((enhanc* or expand*) adj3 role?).tw.

24. 5 or 6 or 7 or 8 or 9 or 10 or 11 or 12 or 13 or 14 or 15 or 16 or 17 or 18 or 19 or 20 or 21 or 22 or 23

25. 4 and 24

26. ((community or primary care or primary health care or primary healthcare) adj3 paramedic*).tw.

27. 25 or 26

28. limit 27 to yr="2001 -Current"

# Appendix B. List of included studies

| **Lead author** | **Year** | **Origin** | **Title** | **Aim of study** | **Evidence category under investigation** | **Study design** | **Population description** | **Number of participants** |
| --- | --- | --- | --- | --- | --- | --- | --- | --- |
| Abrashkin | 2016 | United States | Providing Acute Care at Home: Community Paramedics Enhance an Advanced Illness Management Program-Preliminary Data. | Explored the feasibility of in-home evaluation and treatment of acute illnesses by paramedics within an Advanced Illness Management (AIM) program. | Outcomes from community paramedicine programmes (such as quality of life, patient satisfaction, and economic impact) | Cohort study | Two-thirds were female, the median age was 83, and the median number of ADL dependencies was 5 (range 0-6). The study population had high rates of chronic conditions: dementia (44%), decubitus ulcers (29%), diabetes mellitus (26%), congestive heart failure (24%), and chronic obstructive pulmonary disease (15%) | 1602 |
| Abrashkin | 2019 | United States | Community paramedics treat high acuity conditions in the home: a prospective observational study | Explored whether high acuity conditions that would typically result in transport to the ED in a conventional 911 system can be effectively treated at home using a physician extender CP model within advanced illness management (AIM) programme | Models of delivery to include clinical governance, supervision, and other structural supports; Outcomes from community paramedicine programmes (such as quality of life, patient satisfaction, and economic impact) | Prospective observational | All individuals enrolled in the AIM programme were eligible to receive care through the CP programme. Enrolment criteria for the AIM programme include being home-bound with two or more chronic conditions. | 3137 |
| Adio | 2020 | United States | Community Paramedics' Perception of Frequent ED Users and the Community Paramedicine Program: A Mixed-Methods Study. | Addresses the following research questions: 1. What are the views of paramedics towards frequent emergency department users and underlying causes? 2. What are the views of paramedics about the community paramedicine program related to its relevance and administration, and their personal experiences and professional competencies? | Outcomes from community paramedicine programmes (such as quality of life, patient satisfaction, and economic impact) | Mixed Methods | 16 Community Integrated Health Program paramedics | 16 |
| Agarwal | 2020 | Canada | Cost-effectiveness analysis of a community paramedicine programme for low-income seniors living in subsidised housing: the community paramedicine at clinic programme (CP@clinic). | To evaluate the cost-effectiveness of the CP@clinic programme compared with usual care for low-income seniors living in subsidised (social) housing | Outcomes from community paramedicine programmes (such as quality of life, patient satisfaction, and economic impact) | Economic evaluation | Aged over 55 years of age, living in mid-and high-rise public housing buildings for low-income older adults, where a portion of the rental fees are subsidised by the government | 678 |
| Agarwal | 2017 | Canada | Effectiveness of a community paramedic-led health assessment and education initiative in a seniors' residence building: the Community Health Assessment Program through Emergency Medical Services (CHAP-EMS). | The aim was to evaluate whether a weekly 8-hour CHAP-EMS program was associated with changes in (1) number of emergency EMS calls (9-1-1) from the seniors’ residence building, (2) mean blood pressure (BP) of participants and (3) diabetes risk profile of participants after one year of implementation | Outcomes from community paramedicine programmes (such as quality of life, patient satisfaction, and economic impact) | Non-randomised experimental study | Eligible study participants were apartment building residents over 65 years living in one of 260 apartments units which were identified as having a high volume of ambulance call-outs. Most occupants were assessed as being of low income and, accordingly, were receiving rent subsidies | 79 |
| Agarwal | 2020 | Canada | Feasibility of implementing a community cardiovascular health promotion program with paramedics and volunteers in a South Asian population. | The aim was to assess whether the key components of the CP@clinic program (e.g., paramedic-led sessions, risk assessments, referrals, reports to primary care) can be feasibly implemented in a South Asian community setting. The secondary objective of the study was to describe cardiometabolic risk factors observed in this high-risk population to inform a future full-scale study and health promotion and disease prevention initiatives. | Outcomes from community paramedicine programmes (such as quality of life, patient satisfaction, and economic impact) | Mixed Methods | Participants consisted of adults visiting a local community recreation centre or a local Sikh Temple. Predominantly North Indian, speaking Hindi, Punjabi and Urdu. | 71 |
| Agarwal | 2018 | Canada | Evaluation of a community paramedicine health promotion and lifestyle risk assessment program for older adults who live in social housing: a cluster randomized trial. | The aim was to use a randomized controlled trial (RCT) to determine if implementing CP@clinic decreases mean ambulance calls (primary outcome) in the intervention versus control buildings, measured at the building level. Secondary outcomes were improvement in risk-factor profiles and HRQoL among older adults living in subsidised community housing (individual-level measures and analysis) | Outcomes from community paramedicine programmes (such as quality of life, patient satisfaction, and economic impact) | Randomised controlled trial | Aged over 55 years of age, living in mid-and high-rise public housing buildings for low-income older adults, where a portion of the rental fees are subsidised by the government | 158 |
| Agarwal | 2019 | Canada | Reducing 9-1-1 Emergency Medical Service Calls By Implementing A Community Paramedicine Program For Vulnerable Older Adults In Public Housing In Canada: A Multi-Site Cluster Randomized Controlled Trial. | Evaluate the change in mean EMS calls at the building-level, comparing intervention and control buildings, across multiple community sites. | Outcomes from community paramedicine programmes (such as quality of life, patient satisfaction, and economic impact) | Randomised controlled trial | Aged over 55 years of age, living in mid-and high-rise public housing buildings for low-income older adults, where a portion of the rental fees are subsidized by the government | 1509 |
| Ash | 2020 | United States | Quality of Life for Persons with Chronic Disease Utilizing Mobile Integrated Healthcare | The study aimed to analyse the relationship between NCD type; age; gender; duration of participation in MIH-CP; hospital readmission; and self-reported, perceived QOL as measured by the EQ-5D-3L for those who received services from the MIH-CP program | Outcomes from community paramedicine programmes (such as quality of life, patient satisfaction, and economic impact) | Cohort study | Patients enrolled in MIH- CP program. Predominantly patients with non-communicable diseases over the age of 18 | 645 |
| Ashton | 2017 | Canada | Conserving Quality of Life through Community Paramedics. | To determine whether community paramedicine services (the intervention through home visits) would have a positive economic impact through influencing the self-perceived quality of life and determining a monetised value | Outcomes from community paramedicine programmes (such as quality of life, patient satisfaction, and economic impact) | Economic evaluation | Clients who were high users of healthcare services (greater than 3 occasions of service with EMS) and had one or more of five chronic diseases (congestive heart failure, chronic obstructive pulmonary disease, hypertension, stroke, and diabetes) at 2 sites Renfrew and Hastings | 200 |
| Ball | 2005 | International | Setting the scene for the paramedic in primary care: a review of the literature. | The review explores the published evidence which surrounds paramedic practice in an attempt to identify the skills, training, and professional capacity that paramedics of the future will require | Models of delivery to include clinical governance, supervision, and other structural supports; Outcomes from community paramedicine programmes (such as quality of life, patient satisfaction, and economic impact) | Literature review | N/A | Not reported |
| Batt | 2021 | Canada | Advances in Community Paramedicine in Response to COVID-19 | To investigate how have Canadian community paramedicine programmes innovated in response to COVID-19 and how can these innovations inform the future development of community paramedicine? | Scope of role | Grey literature | N/A | N/A |
| Bennett | 2017 | United States | Community Paramedicine Applied in a Rural Community. | The aim was to determine if the CP program reduced ED visits in Abbeville while improving patient outcomes | Outcomes from community paramedicine programmes (such as quality of life, patient satisfaction, and economic impact) | Non-randomised experimental study | 68 participants in the intervention arm, 15 had hypertension, 5 had diabetes, 5 had COPD or asthma, 5 had other diseases (2 depression, 2 posttraumatic stress disorder, and 1 blind), and 39 had some combination of the above. Participants were 60.3% female, 64.7% non-white, with an average age of 57.6 years and were enrolled an average of 355.3days in the program at the time of analysis | 193 |
| Bennett | 2020 | United States | Community Paramedicine Applied in a Rural Community | The objective of this study was to determine if the CP program reduced ED visits in Abbeville while improving patient outcomes | Outcomes from community paramedicine programmes (such as quality of life, patient satisfaction, and economic impact) | Pre/post-test with a comparison group study design | CP patients in a rural US county. Eligible for CP program if they have >2 ED visits within a month. Minimum of 1 chronic disease and were frequent users of other health care services. | 193 (n=68 enrolled vs. n=125 comparison) |
| Bigham | 2013 | Canada | Expanding paramedic scope of practice in the community: a systematic review of the literature. | Review of the international literature to describe existing community paramedic programmes | Models of delivery to include clinical governance, supervision, and other structural supports | Systematic review | N/A | 11 studies |
| Boykin | 2018 | United States | Interprofessional care collaboration for patients with heart failure. | The aim was to describe the process of collaboration among healthcare professionals during TOC from an institution to the home setting. | Models of delivery to include clinical governance, supervision, and other structural supports; Scope of role | Cohort study | Patients with heart failure | N/A |
| Bradley | 2016 | United States | The business case for community paramedicine: lessons from Commonwealth Care Alliances Pilot Program | Summarises acute community care program business case | Models of delivery to include clinical governance, supervision, and other structural supports; Outcomes from community paramedicine programmes (such as quality of life, patient satisfaction, and economic impact) | Grey literature | N/A | N/A |
| Brydges | 2014 | Canada | A Case Study of Older Adult Experiences with a Novel Community Paramedicine Program | Aim to understand older adults' experiences with a novel community paramedicine program, the Cardiovascular Health Awareness Program by EMS (CHAP-EMS), operating in a subsidised housing building in Hamilton. | Outcomes from community paramedicine programmes (such as quality of life, patient satisfaction, and economic impact) | Qualitative research | Older adults living in subsidised housing buildings in Hamilton. Age range 63-89. 12/15 lived alone. All lived with a medical problem | 15 |
| Brydges | 2016 | Canada | The CHAP-EMS health promotion program: a qualitative study on participants' views of the role of paramedics. | This study sought to understand participants' perceptions of paramedic providers to explore their role in this unique practice setting and ultimately create an emerging framework to examine paramedic roles in community paramedicine programmes. | Outcomes from community paramedicine programmes (such as quality of life, patient satisfaction, and economic impact) | Qualitative research | Adults living in the residential building in which CHAP-EMS took place including those who were currently participating in the CHAP-EMS program. Aged 63-89, most living alone 12/15, 6/15 males and all had at least one medical problem | 15 |
| Castillo | 2016 | United States | Mobile Integrated Healthcare: Preliminary Experience and Impact Analysis with a Medicare Advantage Population | Aims to describe and analyse the initial experience and preliminary impact of an MIH intervention delivered at scale for a high-risk subpopulation. | Outcomes from community paramedicine programmes (such as quality of life, patient satisfaction, and economic impact) | Cohort study | Conducted utilising the experience and data from an MIH care coordination program for the state-wide membership of a Medicare Advantage Preferred Provider Organisation (MAPPO) population in Florida. Consisted of complex and vulnerable patients in the home and alternative settings | 1074 (intervention) 1241 (control) |
| Chan | 2019 | International | Community paramedicine: A systematic review of program descriptions and training. | A systematic review was conducted to 1) identify the key differences between community paramedicine programmes for program classification, and 2) describe the training required for each program type | Education (including entry-level requirements) | Systematic review | N/A | 64 studies |
| Choi | 2016 | United States | Mobile Integrated Health Care and Community Paramedicine: An Emerging Emergency Medical Services Concept. | Literature review of MIH CP | Education (including entry-level requirements); Outcomes from community paramedicine programmes (such as quality of life, patient satisfaction, and economic impact) | Systematic review | N/A | Not reported |
| Clarke | 2019 | United Kingdom | What are the clinical practice experiences of specialist and advanced paramedics working in emergency department roles? A qualitative study. | This study aimed to explore the lived experiences of paramedics who have made the transition from the ambulance service to specialist/advanced ED roles in the United Kingdom, and to explore how working in this new clinical environment influenced their clinical practice | Outcomes from community paramedicine programmes (such as quality of life, patient satisfaction, and economic impact) | Qualitative research | Qualified paramedics working within an ED (4-24 months experience) | 8 |
| Constantine | 2021 | United States | Implementation of Drive-through Testing for COVID-19 with Community Paramedics | Aimed to describe the Implementation of Drive-through Testing for COVID-19 with Community Paramedics | Models of delivery to include clinical governance, supervision, and other structural supports | Cohort study | Anyone with concerns  about being exposed to, or having the symptoms of, COVID-19 could be screened for testing. | 4342 |
| Cooper | 2004 | United Kingdom | The emerging role of the emergency care practitioner | To examine the emerging role of the emergency care practitioner (ECP) with comparisons to paramedic practice. | Models of delivery to include clinical governance, supervision, and other structural supports; Outcomes from community paramedicine programmes (such as quality of life, patient satisfaction, and economic impact) | Mixed Methods | 15 paramedics (4 ECP 11 paramedics) | 692 cases attended. Paramedics reported on 331 cases while the ECPs reported on 170 cases within the EMS system and 191 MIU cases. |
| Cooper | 2007 | United Kingdom | Collaborative practices in unscheduled emergency care: role and impact of the emergency care practitioner--qualitative and summative findings. | Aimed to investigate the ECP role and collaborative experiences. | Outcomes from community paramedicine programmes (such as quality of life, patient satisfaction, and economic impact) | Mixed Methods | Senior health authority and trust managers, A&E consultants and senior nurses, paramedics, general practitioners (GPs) and practice managers, care home managers, social services and Falls group leads. | 21 |
| Counts | 2017 | United States | An Evaluation of the Environmental and Organizational Factors Associated with the Formation of Community Paramedicine Programmes | To evaluate the environmental and organisational characteristics that foster the existence of MIHCP programmes and aimed to determine if those counties with programmes see a systematically measurable effect on related EMS call volumes | Models of delivery to include clinical governance, supervision, and other structural supports | Cross sectional study | N/A | N/A |
| Dainty | 2018 | Canada | Home Visit-Based Community Paramedicine and Its Potential Role in Improving Patient-Centered Primary Care: A Grounded Theory Study and Framework. | To understand the experiences and perspectives of patients and families involved with the Expanding Paramedicine in the Community (EPIC) randomised trial in Ontario, Canada, as well as how such a model is shaped and enabled according to the needs and interests of programme participants | Education (including entry-level requirements); Outcomes from community paramedicine programmes (such as quality of life, patient satisfaction, and economic impact) | Qualitative research | Patients who had received at least three scheduled visits from the EPIC community paramedic | 40 |
| Dixon | 2008 | United Kingdom | Is it cost effective to introduce paramedic practitioners for older people to the ambulance service? Results of a cluster randomised controlled trial | To assess the cost-effectiveness of the paramedic practitioner (PP) scheme compared with usual emergency care. | Outcomes from community paramedicine programmes (such as quality of life, patient satisfaction, and economic impact) | Randomised controlled trial | Patients >60 years old who presented to ambulance service in an urban setting | 3018 |
| Eaton | 2021 | United Kingdom | Designing and implementing an educational framework for advanced paramedic practitioners rotating into primary care in North Wales | Aimed to evaluate an educational framework to determine how it supported the development of Advanced Paramedic Practitioners in the primary care setting | Education (including entry-level requirements) | Evaluation/ qualitative | Advanced Paramedic Practitioners rotating through primary care, and GPs offering supervision. | 11 (7 Advanced Paramedics; 4 GPs) |
| Eaton | 2021 | International | Understanding the role of the paramedic in primary care: a realist review. | To understand how paramedics impact (or not) the primary care workforce | Education (including entry-level requirements); Models of delivery to include clinical governance, supervision, and other structural supports; Outcomes from community paramedicine programmes (such as quality of life, patient satisfaction, and economic impact); Scope of role | Realist Review | N/A | 205 articles |
| Eaton | 2020 | United Kingdom | Contribution of paramedics in primary and urgent care: a systematic review. | To describe gaps in the current UK literature regarding the work of paramedics in primary care | Education (including entry-level requirements); Models of delivery to include clinical governance, supervision, and other structural supports; Outcomes from community paramedicine programmes (such as quality of life, patient satisfaction, and economic impact); Scope of role | Systematic review | N/A | 14 studies |
| Evans | 2012 | United Kingdom | Which extended paramedic skills are making an impact in emergency care and can be related to the UK paramedic system? A systematic review of the literature. | To identify evidence of paramedics trained with extra skills and the impact of this on patient care and interrelating services such as General Practices or Emergency Departments | Scope of role | Systematic review | N/A | 19 studies |
| Feldman | 2021 | United States | "House Calls" by Mobile Integrated Health Paramedics for Patients with Heart Failure: A Feasibility Study | To assess the feasibility of integrating community paramedics into the out-patient management of patients with heart failure with scheduled urgent house calls | Models of delivery to include clinical governance, supervision, and other structural supports | Cohort study | Patients >18 years with acute or acute-on-chronic heart failure, in the geographical boundary of EMS | 40 |
| Flint | 2019 | United States | The Systemic Impacts of Integrated Mobile Healthcare in a State-wide Emergency Medical Services System | To review the impact of IMHC programmes on transport requests, operational surges and fiscal loss of the EMS providing the programme | Models of delivery to include clinical governance, supervision, and other structural supports | Mixed method literature review, retrospective data analysis | N/A | 4529 CP calls |
| Ford-Jones | 2020 | Canada | Filling the gap: Mental health and psychosocial paramedicine programming in Ontario, Canada | To identify promising programmes for paramedics attending calls for mental health needs in Ontario, Canada. | Scope of role | Qualitative research | N/A | N/A |
| Gingold | 2021 | United States | The effect of a mobile integrated health program on health care cost and utilization. | To measure the effect of a mobile integrated health community paramedicine (MIH-CP) transitional care program on hospital utilisation, emergency department visits, and charges | Outcomes from community paramedicine programmes (such as quality of life, patient satisfaction, and economic impact) | Cohort study | Patients over 18 years old, discharged to home from internal/family medicine services | 464 |
| Glenn | 2017 | United States | State Regulation of Community Paramedicine Programmes: A National Analysis. | Aimed to examine the current scope of practice related to CP, as defined by state statutes and regulations in the 50 U.S. states | Models of delivery to include clinical governance, supervision, and other structural supports | Systematic review | N/A | N/A |
| Goldberg | 2014 | United States | Mobile integrated healthcare: Using existing out of hospital resources to bridge gaps in healthcare services | Aimed to examine how current EMS systems might bridge the gap between at-risk patient populations and health care services through MIH programmes | Outcomes from community paramedicine programmes (such as quality of life, patient satisfaction, and economic impact) | Systematic review | N/A | Not reported |
| Gregg | 2019 | United States | Systematic Review of Community Paramedicine and EMS Mobile Integrated Health Care Interventions in the United States. | To determine the effectiveness of CP-MIH interventions at addressing the Quadruple Aim (which focuses on controlling health care costs while improving population health and both provider and patient satisfaction) | Outcomes from community paramedicine programmes (such as quality of life, patient satisfaction, and economic impact) | Systematic review | N/A | 8 studies |
| Halter | 2006 | United Kingdom | Patients' experiences of care provided by emergency care practitioners and traditional ambulance practitioners: A survey from the London Ambulance Service | To compare patient experiences of ECP care with that from traditional ambulance practitioners (state-registered paramedic or emergency medical technician). | Outcomes from community paramedicine programmes (such as quality of life, patient satisfaction, and economic impact) | Survey | No details given | 888 |
| Hanninen | 2020 | Finland | Patients Seeking Retreatment after Community Paramedic Assessment and Treatment: Piloting a Community Paramedic Unit Program in Southwest Finland | To categorise CP unit patients seeking retreatment after a CP unit visit and investigate links between CP unit actions and patients seeking retreatment. | Outcomes from community paramedicine programmes (such as quality of life, patient satisfaction, and economic impact) | Retrospective analysis | Not reported | 229 |
| Harvey | 2021 | United Kingdom | The ambulance service advanced practitioner's role in supporting care homes: a qualitative study of care staff experiences | Investigated the experiences and needs of the care home staff who use the ambulance service, advanced practitioner model. | Outcomes from community paramedicine programmes (such as quality of life, patient satisfaction, and economic impact) | Qualitative research | Staff members from 10 different care homes | 19 |
| Hill | 2013 | United Kingdom | A systematic review of the activity and impact of emergency care practitioners in the NHS. | Summarise the impact of ECPs on healthcare delivery and effectiveness as a health service resource | Outcomes from community paramedicine programmes (such as quality of life, patient satisfaction, and economic impact) | Systematic review | N/A | 5 studies |
| Hoyle | 2012 | New Zealand | Introduction of an extended care paramedic model in New Zealand | The study aimed to determine the rate of treatment in the community and to examine any acute hospital presentation within 7 days from ECP presentation. | Outcomes from community paramedicine programmes (such as quality of life, patient satisfaction, and economic impact) | Cohort study | The Kapiti population was just under 50 000 people in 2009. The district is a popular retirement area, and as a result, the proportion of residents over the age of 65 is twice the national average. The most common presentations were falls and respiratory problems, accounting for 13% and 9.8% of presentations, respectively. | 1000 |
| Hughes | 2021 | United Kingdom | Community paramedicine home visits: patient perceptions and experiences | To explore patient perceptions and experiences of CP home visits delivered by specialist paramedics (SPs) in a Scottish urban general practice home-visit setting. | Outcomes from community paramedicine programmes (such as quality of life, patient satisfaction, and economic impact) | Case series | Adults who have mental capacity and have received at least one SP home visit on behalf of their general practice | 16 |
| Jensen | 2016 | Canada | Impact of a Novel Collaborative Long-Term Care EMS Model: A Before-and-After Cohort Analysis of an Extended Care Paramedic Program | The objective of this study was to measure differences in the delivery of emergency care for LTC residents with acute illnesses or injuries attended by ECP or emergency paramedics, measured primarily with a number of transports to the ED, as well as EMS response and scene time, patient ED length of stay, EMS time in the ED, hospital admission, and relapse back to EMS after calls ending in no transport. | Outcomes from community paramedicine programmes (such as quality of life, patient satisfaction, and economic impact) | Cohort study | Residents of long-term care | 360 |
| Kant | 2018 | United States | Outcomes and provider perspectives on geriatric care by a nurse practitioner-led community paramedicine program. | The aim was to describe patients, outcomes, and geriatric primary care provider perspectives related to the use of a community paramedicine program. | Outcomes from community paramedicine programmes (such as quality of life, patient satisfaction, and economic impact) | Case series | Geriatric patients | 40 |
| Keefe | 2020 | United States | Behavioural Health Emergencies Encountered by Community Paramedics: Lessons from the Field and Opportunities for Skills Advancement. | To examine paramedics perceptions and experiences responding to behavioural health crises in the USA | Scope of role | Qualitative research | Paramedics working in the Acute Community Care Programme | 23 |
| Knowles | 2010 | United Kingdom | An initiative to provide emergency healthcare for older people in the community: the impact on carers. | Describe the impact of a new model of service delivery on the carers/support persons for patients that received care. | Outcomes from community paramedicine programmes (such as quality of life, patient satisfaction, and economic impact) | Cross-sectional study | Individuals that provided physical or emotional support to a patient and was present when paramedic practitioners provided care. | 569 |
| Lau | 2018 | United States | Qualitative Evaluation of the Coach Training within a Community Paramedicine Care Transitions Intervention. | Aimed to define community paramedics perceptions regarding their training needs to serve as Care Transitions Intervention (CTI) coaches supporting the ED-to-home transition | Education (including entry-level requirements) | Qualitative research | Participants were identified from a list of active community paramedics currently acting as CTI coaches in Madison, Wisconsin and Rochester, New York. Participants consisted solely of non-Hispanic whites included five women, and had a mean age of 43. Participants had extensive backgrounds in healthcare, primarily as EMS providers, but minimal experience with community paramedicine. All reported some prior geriatrics training. | 8 |
| Leduc | 2020 | International | The Safety and Effectiveness of On-Site Paramedic and Allied Health Treatment Interventions Targeting the Reduction of Emergency Department Visits by Long-Term Care Patients: Systematic Review. | A systematic review of the literature to determine, among long-term care patients, what is the effectiveness and safety of interventions that evaluate and treat patients on-site, avoiding unscheduled transport to the ED. | Outcomes from community paramedicine programmes (such as quality of life, patient satisfaction, and economic impact) | Systematic review | N/A | 22 studies |
| Leyenaar | 2021 | Canada | Relevance of assessment items in community paramedicine home visit programmes: results of a modified Delphi study. | To investigate the relevance of assessment items to the practice of community paramedics according to a pre-established clarity-utility matrix | Scope of role | Delphi | The expert panel of community paramedics from one Canadian province | 26 |
| Leyenaar | 2019 | Canada | Examining consensus for a standardised patient assessment in community paramedicine home visits: a RAND/UCLA-modified Delphi Study. | Investigate the level of consensus that could be found by a panel of experts regarding appropriate health, social and environmental domains that should be assessed in community paramedicine home visit programme | Education (including entry-level requirements) | Delphi | Patients served by community paramedicine programmes | 17 |
| Leyenaar | 2019 | Canada | What do community paramedics assess? An environmental scan and content analysis of patient assessment in community paramedicine. | Aimed to summarise the content of assessment instruments and describe the state of current practice in community paramedicine home visit programmes | Scope of role | Environmental scan | Ontario Community Paramedic programmes | 43 |
| Leyenaar | 2019 | Canada | Report on the status of community paramedicine in Ontario | Report summarising community paramedicine in Ontario | Models of delivery to include clinical governance, supervision, and other structural supports; Outcomes from community paramedicine programmes (such as quality of life, patient satisfaction, and economic impact); Scope of role | Grey literature | In 2018-19, 39 (75%) Ontario’s municipal paramedic services were delivering more than one community paramedicine model of care. 35 (90%) were offering Home Visit Programmes that served an estimated 3,790 patients, 23 (59%) were offering Community Paramedic-Led Clinics that served an estimated 17,680 patients, 23 (59%) were offering Remote Patient Monitoring programmes that served an estimated 2,300 patients, 10 (26%) were offering Community Paramedic-Specialist Response Programmes that served an estimated 7,840 patients, and 6 (15%) were offering additional programmes that served another 1,990 patients. | N/A |
| Leyenaar | 2018 | Canada | A scoping study and qualitative assessment of care planning and case management in community paramedicine | The objective of this study is to contribute to paramedic practice by examining broad areas of care planning in CP, identifying gaps in the evidence, clarifying key concepts, and reporting on the types of evidence that address and inform practice. | Models of delivery to include clinical governance, supervision, and other structural supports; Other | Qualitative research | N/A | 10 studies included |
| Leyenaar | 2021 | Canada | Paramedics assessing patients with complex comorbidities in community settings: results from the CARPE study. | This study aimed to provide information about how community paramedicine home visit programmes best navigate their role in delivering preventative care to frequent 9-1-1 users by describing the demographic and clinical characteristics of their patients and comparing them to existing community care populations. | Outcomes from community paramedicine programmes (such as quality of life, patient satisfaction, and economic impact) | Qualitative research | Home care clients, community support services clients and community paramedicine clients. | n=43,856 (29,938 home care clients, 13,782 community support services clients, and 136 community paramedicine patients) |
| Lezzoni | 2018 | United States | Early experiences with the Acute Community Care Program in eastern Massachusetts. | To describe experiences during the first 2 years of the Acute Community Care Program (ACCP) | Outcomes from community paramedicine programmes (such as quality of life, patient satisfaction, and economic impact) | Mixed methods | Socioeconomically disadvantaged adults with complex health needs | 601 |
| Martin | 2016 | Canada | Consumer perspectives of a community paramedicine program in rural Ontario. | The aim was to report on a community paramedicine program in rural Ontario, Canada, through the perceptions and experiences of consumers | Outcomes from community paramedicine programmes (such as quality of life, patient satisfaction, and economic impact) | Qualitative research | Community members (patients, relatives and carers) referred to as consumers, all were Caucasian Canadians. | 14 |
| Martin | 2019 | Canada | Perspectives from the frontline of two North American community paramedicine programmes: an observational, ethnographic study. | The purpose of this study was to identify the motivations, job satisfaction and challenges of community paramedics | Models of delivery to include clinical governance, supervision, and other structural supports | Qualitative research | Paramedic service managers and community paramedics from two paramedic services. | 15 |
| Martin-Misener | 2009 | Canada | Cost effectiveness and outcomes of a nurse, practitioner paramedic, family physician model of care: the Long and Brier Islands study | To assess if patients in the collaborative model demonstrate evidence of improved psychosocial adjustment and less expenditure of health care resources over time | Outcomes from community paramedicine programmes (such as quality of life, patient satisfaction, and economic impact) | Economic evaluation | Adult English-speaking permanent residents of the Islands, age 40 years or more with a diagnosis of at least one chronic illness, aware of their diagnosis and able to provide informed written consent | 221 |
| Mason | 2007 | United Kingdom | Effectiveness of emergency care practitioners working within existing emergency service models of care | Aimed to (1) evaluate appropriateness, satisfaction and cost of ECPs compared with the usual service available in the same healthcare setting, (2) to increase understanding of what effect, if any, ECPs are having on the delivery of health services locally and (3) to evaluate whether ECP working yields cost savings. | Models of delivery to include clinical governance, supervision, and other structural supports | Cross-sectional study | Patients who were seen by an ECP | 524 |
| Mason | 2007 | United Kingdom | Effectiveness of paramedic practitioners in attending 999 calls from elderly people in the community: cluster randomised controlled trial. | To evaluate the benefits of paramedic practitioners assessing and, when possible, treating older people in the community after minor injury or illness. Paramedic practitioners have been trained with extended skills to assess, treat, and discharge older patients with minor acute conditions in the community. | Outcomes from community paramedicine programmes (such as quality of life, patient satisfaction, and economic impact) | Randomised controlled trial | patients aged over 60 years who called the emergency services within the study period (Sept. 2003-Sept 2004) from a Sheffield postal code, between the hours of 8am-8pm with a presenting complaint that fell within the paramedic practitioner scope of practice | 3018 (n=1549 intervention vs. n=1469 control) |
| Mason | 2008 | United States | Safety of paramedics with extended skills | Aimed to evaluate the safety of clinical decisions made by Paramedic Practitioners operating within the new service. | Outcomes from community paramedicine programmes (such as quality of life, patient satisfaction, and economic impact) | Randomised controlled trial | Patients aged >60 years contacting the emergency medical services (EMS) with a minor injury or illness were included in the study. | 3,018 (n=1549 intervention vs. n=1469 control) |
| Misra-Hebert | 2021 | United States | Healthcare utilization and patient and provider experience with a home visit program for patients discharged from the hospital at high risk for readmission. | Aimed to assess the association with health care utilisation and mortality for patients at high risk for readmission who participated in the post-discharge home visit program and to examine provider and patient experience regarding program participation. | Outcomes from community paramedicine programmes (such as quality of life, patient satisfaction, and economic impact) | Mixed Methods | Adult patients with a Cleveland Clinic Health System Primary Care Provider following hospital discharge | Varied according to methods of analysis |
| Nejtek | 2017 | United States | A pilot mobile integrated healthcare program for frequent utilizers of emergency department services | Aimed to examine whether or not a mobile integrated health (MIH) program may improve health-related quality of life while reducing emergency department (ED) transports, ED admissions, and inpatient hospital admissions in frequent utilisers of ED services | Outcomes from community paramedicine programmes (such as quality of life, patient satisfaction, and economic impact) | Cohort study | Participants in a pilot program in North Texas, USA | 64 |
| Nowrouzi-Kia | 2021 | Canada | Quality of work life of paramedics practicing community paramedicine in northern Ontario, Canada: a mixed-methods sequential explanatory study | To evaluate several pilot CP programmes in northern Ontario from the perspectives of paramedics, to gain program recommendations related to both rural and urban settings across northern Ontario | Outcomes from community paramedicine programmes (such as quality of life, patient satisfaction, and economic impact) | Cross-sectional study | Community Paramedics | 75 |
| O'Meara | 2003 | Australia | Would a prehospital practitioner model improve patient care in rural Australia? | Aimed to develop and critically appraise the prehospital practitioner model as an alternative to existing models in rural Australia | Models of delivery to include clinical governance, supervision, and other structural supports; Outcomes from community paramedicine programmes (such as quality of life, patient satisfaction, and economic impact) | Soft systems methodology | N/A | N/A |
| O'Meara | 2015 | Canada | Integrating a community paramedicine program with local health, aged care and social services: An observational ethnographic study | To identify and describe the nature of the relationship between public engagement and the integration of CP with local health, aged care and social services | Models of delivery to include clinical governance, supervision, and other structural supports | Qualitative research | Paramedic service managers, paramedics, educators, physicians, nurses, other health professionals, patients and community members. involved with community paramedicine programming in a rural Canadian setting. | Focus groups, interviews, and field observations. |
| O'Meara | 2016 | Canada | Community paramedicine model of care: an observational, ethnographic case study. | The study aimed to identify and analyse how community paramedics create and maintain new role boundaries and identities in terms of flexibility and permeability and through this develop and frame a coherent community paramedicine model of care that distinguishes the model from other innovations in paramedic service delivery | Models of delivery to include clinical governance, supervision, and other structural supports | Qualitative research | Community members, including patients, family and carers, Paramedics and paramedic service managers from Renfrew County and the Greater Ottawa area, Paramedicine educators in Ontario, Physicians, nurse practitioners and other health care providers who interact with community paramedics, Health economists and health service managers | up to 94 |
| Pang | 2019 | International | Limited data to support improved outcomes after community paramedicine intervention: A systematic review. | The objective was to systematically review the literature to describe the outcomes utilised by CP programmes and the extent to which CP programmes improved those outcomes | Outcomes from community paramedicine programmes (such as quality of life, patient satisfaction, and economic impact) | Systematic review | N/A | 6 studies |
| Pearson | 2014 | United States | The Evidence for Community Paramedicine in Rural Areas: State and local findings and the role of the state Flex program | Examined the evidence base for community paramedicine in rural communities, the role of community paramedics in rural healthcare delivery systems, the challenges faced by states in implementing community paramedicine programmes, and the role of the state Flex programmes in supporting the development of community paramedicine programmes. | Education (including entry-level requirements); Models of delivery to include clinical governance, supervision, and other structural supports | Survey exercise and literature review | N/A | N/A |
| Pearson | 2017 | United States | Community Paramedicine pilot programmes: lessons from Maine | Aimed to describe the healthcare needs of people living in rural areas and of how community paramedicine can address some of those needs. | Models of delivery to include clinical governance, supervision, and other structural supports | Qualitative research | N/A | N/A |
| Proctor | 2019 | United Kingdom | Home visits from paramedic practitioners in general practice: patient perceptions | To explore older patients’ perceptions of having PPs, who work in GP surgeries, attend to them on a home visit in place of the GP. | Outcomes from community paramedicine programmes (such as quality of life, patient satisfaction, and economic impact) | Qualitative research | Patients aged 65 or older who had called the GP surgery in hours asking for a GP to visit. The six participants included three females and three males, aged 77-88 years, all of the white British ethnicity. | 6 |
| Quatman-Yates | 2021 | United States | Assessment of Fall-Related Emergency Medical Service Calls and Transports After a Community-Level Fall-Prevention Initiative | Investigated the impact of a community paramedic programmes optimisation of a fall prevention system entailing a clinical pathway and learning health system (called Community-FIT) on community-level fall-related emergency medical service utilisation rates | Outcomes from community paramedicine programmes (such as quality of life, patient satisfaction, and economic impact) | Prevalence study | A midwestern suburban city in the USA | 892 fall-related call outs |
| Rasku | 2019 | Finland | The core components of Community Paramedicine - integrated care in primary care setting: a scoping review. | This scoping review aimed to describe and analyse published empirical studies and program reports describing Community Paramedicine (CP)to find out the core components of CP. | Models of delivery to include clinical governance, supervision, and other structural supports | Systematic review | N/A | 21 studies |
| Raynovich | 2014 | United States | A survey of community paramedicine course offerings and planned offerings | The feedback received from the respondents has helped to inform the curriculum development group about possible changes that can be made to improve the curriculum. | Education (including entry-level requirements) | Survey | The respondents included administrative and educator representatives of accredited post-secondary educational institutions and Government officials, all of whom had previously requested a copy of the curriculum. As such, this was a population survey and not a sample. | A total of 223 surveys were sent out and 68 (30.49%) responses were received. |
| Ritchie | 2020 | United States | Readiness of Stakeholders to Adopt Community Paramedicine Programmes in Tennessee | Aimed to explore opinions, attitudes, and beliefs among key policymakers regarding the adoption of community paramedicine programmes in Tennessee | Outcomes from community paramedicine programmes (such as quality of life, patient satisfaction, and economic impact) | Qualitative research | EMS directors or other officials, physicians, county mayors, and home health representatives | 21 |
| Ruest | 2017 | Canada | Community health evaluation completed using paramedic service (CHECUPS): design and implementation of a new community based health program | Overview of design and evaluation of the implementation of CHECUPS program | Education (including entry-level requirements); Models of delivery to include clinical governance, supervision, and other structural supports; Outcomes from community paramedicine programmes (such as quality of life, patient satisfaction, and economic impact) | Case report | Patients who "struggle with multiple complex and often interrelated health and social care issues | 222 |
| Ruest | 2012 | Canada | Evaluating the impact on 911 calls by an in-home programme with a multidisciplinary team | Review whether the use of community paramedics as part of integrated health care teams can reduce 911 calls | Outcomes from community paramedicine programmes (such as quality of life, patient satisfaction, and economic impact) | Case series | On the waiting list for LTC, able to remain at home until then | 27 |
| Scharf | 2017 | United States | Mobile Integrated Community Health Pilot Program Descriptive Study: Diagnosis Prevalence and Comorbidity among Program Participants | Aimed to evaluate the prevalence of disease among patients in a MIH-CP pilot program | Models of delivery to include clinical governance, supervision, and other structural supports | Prevalence study | Queen Anne’s County Mobile Integrated Community Healthcare Pilot Program participants | 97 |
| Schwab-Reese | 2021 | United States | "They're very passionate about making sure that women stay healthy": a qualitative examination of women's experiences participating in a community paramedicine program. | Aimed to evaluate women’s experiences in Project Swaddle | Outcomes from community paramedicine programmes (such as quality of life, patient satisfaction, and economic impact) | Qualitative research | Pregnant and postpartum women and their infants. | 15 |
| Seidl | 2021 | United States | Development of a Logic Model to Guide Implementation and Evaluation of a Mobile Integrated Health Transitional Care Program. | This paper describes the structured process for developing a logic model. | Models of delivery to include clinical governance, supervision, and other structural supports | Retrospective analysis | Any patient from the inpatient, observation, or ED setting who lives within the 6 eligible zip codes | 450 approached, approx. 75% acceptance rate |
| Shah | 2018 | United States | Improving the ED-to-Home Transition: The Community Paramedic-Delivered Care Transitions Intervention-Preliminary Findings. | Aimed to describe the community paramedic-delivered care transitions intervention preliminary findings. | Outcomes from community paramedicine programmes (such as quality of life, patient satisfaction, and economic impact) | Randomised controlled trial | ED patients aged > 60 years receiving discharge home are approached and, if consenting to participate, randomized to the CTI Program or usual car. | 853 |
| Siddle | 2017 | United States | Mobile integrated health to reduce post-discharge acute care visits: A pilot study. | Aimed to evaluate the efficacy of a MIH led transitional care strategy to reduce acute care utilisation. | Outcomes from community paramedicine programmes (such as quality of life, patient satisfaction, and economic impact) | Cohort study | Targeted patients suffering from Chronic Obstructive Pulmonary Disease (COPD), Pneumonia (PNA), Myocardial Infarction (MI) and Heart Failure (HF) before discharge | 203 |
| Stirling | 2007 | Australia | Engaging rural communities in health care through a paramedic expanded scope of practice. | Aims to contribute to understanding how ESP can improve rural health services through successful collaborations | Scope of role | Qualitative research | N/A | 17 |
| Swain | 2012 | New Zealand | Patient satisfaction and outcome using emergency care practitioners in New Zealand. | The purpose of this study was to determine whether patients found the UCC model of service both acceptable and effective and to ascertain whether there was any difference in satisfaction with the care provided by the two groups of paramedics, EAS or ECP. | Outcomes from community paramedicine programmes (such as quality of life, patient satisfaction, and economic impact) | Cross-sectional study | Patients cared for by ECPs | 100 |
| Tangherlini | 2016 | United States | The HOME Team: Evaluating the Effect of an EMS-based Outreach Team to Decrease the Frequency of 911 Use Among High Utilizers of EMS. | Aimed to examine the effectiveness of the HOME project to reduce repeat use of EMS. | Models of delivery to include clinical governance, supervision, and other structural supports | Retrospective analysis | Patients identified as frequent callers of EMS through a monthly list | 508 |
| Thirumalai | 2021 | United States | Challenges and Lessons Learned from a Telehealth Community Paramedicine Program for the Prevention of Hypoglycaemia: Pre-Post Pilot Feasibility Study | Aimed to describe the results of the feasibility evaluation, implementation challenges, and the lessons learned about the deployment of a hypoglycaemia prevention program in an under-served area | Outcomes from community paramedicine programmes (such as quality of life, patient satisfaction, and economic impact) | Single-arm pre/post-test | All patients who called 911 due to hypoglycaemia-related events and met inclusion criteria | 40 |
| Thompson | 2014 | Australia | HWA Expanded Scopes of Practice program evaluation: Extending the Role of Paramedics sub-project | Report on the Extended Care Paramedic program in originally developed in South Australia Ambulance Service and implemented within 5 sites in New South Wales | Education (including entry-level requirements); Models of delivery to include clinical governance, supervision, and other structural supports; Outcomes from community paramedicine programmes (such as quality of life, patient satisfaction, and economic impact); Scope of role | Grey literature | N/A | N/A |
| Thurman | 2021 | International | A scoping review of community paramedicine: evidence and implications for interprofessional practice. | The purpose of this scoping review was to understand the evidence base of CP to inform the further evolution of this model of care. | Models of delivery to include clinical governance, supervision, and other structural supports | Scoping review | N/A | 29 articles |
| Tohira | 2013 | International | The impact of new prehospital practitioners on ambulance transportation to the emergency department: a systematic review and meta-analysis. | To conduct a systematic review and meta-analysis that examined the impact of new prehospital practitioners, including Emergency Care Practitioners (EmCPs), Paramedic Practitioners and Extended Care Paramedics (ECPs) on Emergency Department (ED) services. | Outcomes from community paramedicine programmes (such as quality of life, patient satisfaction, and economic impact) | Systematic review | N/A | 20 studies |
| van Vuuren | 2021 | International | Reshaping healthcare delivery for elderly patients: the role of community paramedicine; a systematic review. | Aimed to identify evidence of the community paramedicine role in care delivery for elderly patients, with an additional focus on palliative care, and the possible impact of this role on the wider healthcare system | Models of delivery to include clinical governance, supervision, and other structural supports | Systematic review | N/A | 13 studies |
| Whalen | 2018 | Canada | The novel role of paramedics in collaborative emergency centres aligns with their professional identity: A qualitative analysis. | Ascertain the attitudes, feelings and experiences of paramedics working within the Nova Scotia Collaborative Emergency Centre construct within an interdisciplinary team | Models of delivery to include clinical governance, supervision, and other structural supports | Qualitative research | Paramedic clinicians providing care through the Collaborative Emergency Centre (CEC) | 14 |
| Widiatmoko | 2008 | United Kingdom | Developing a new response to non-urgent emergency calls: evaluation of a nurse and paramedic partnership intervention | To investigate the Fcost-effectiveness of a new service development whereby a nurse and a paramedic working in partnership attended non-urgent emergency calls | Models of delivery to include clinical governance, supervision, and other structural supports; Outcomes from community paramedicine programmes (such as quality of life, patient satisfaction, and economic impact) | Economic evaluation | Patients involved with non-urgent emergency calls | 2781 |
| Xi | 2021 | International | Paramedics working in general practice: a scoping review | Aimed to identify key issues and gaps to inform follow-on research considering paramedics working in general practice in Ireland | Scope of role | Scoping Study | N/A | 11 articles |
| Xie | 2021 | Canada | Economic Analysis of Mobile Integrated Health Care Delivered by Emergency Medical Services Paramedic Teams. | To compare time on task and cost between MIH and ambulance delivered by NEMS from a public payer’s perspective | Outcomes from community paramedicine programmes (such as quality of life, patient satisfaction, and economic impact) | Economic evaluation | Patients accessing paramedic services in Niagara Region, Ontario, Canada | 6959 calls |

Appendix C

*MMAT Quality assessment results- Qualitative studies*

| **Lead author** | **Year** | **Title** | **Study design** | **Are there clear research questions?** | **Do the collected data allow to address the research questions?** | **Qualitative** | **Is the qualitative approach appropriate to answer the research question?** | **Are the qualitative data collection methods adequate to address the research question?** | **Are the findings adequately derived from the data?** | **Is the interpretation of results sufficiently substantiated by data?** | **Is there coherence between qualitative data sources, collection, analysis and interpretation** |
| --- | --- | --- | --- | --- | --- | --- | --- | --- | --- | --- | --- |
| Brydges | 2014 | A Case Study of Older Adult Experiences with a Novel Community Paramedicine Program | Qualitative research | Yes | Yes |  | Yes | Yes | Yes | Yes | Yes |
| Brydges | 2016 | The CHAP-EMS health promotion program: a qualitative study on participants' views of the role of paramedics. | Qualitative research | Yes | Yes |  | Yes | Yes | Yes | Yes | Yes |
| Clarke | 2019 | What are the clinical practice experiences of specialist and advanced paramedics working in emergency department roles? A qualitative study. | Qualitative research | Yes | Yes |  | Yes | Yes | Yes | Yes | Yes |
| Dainty | 2018 | Home Visit-Based Community Paramedicine and Its Potential Role in Improving Patient-Centered Primary Care: A Grounded Theory Study and Framework. | Qualitative research | Yes | Yes |  | Yes | Yes | Yes | Yes | Yes |
| Ford-Jones | 2020 | Filling the gap: Mental health and psychosocial paramedicine programming in Ontario, Canada | Qualitative research | Yes | Yes |  | Yes | Yes | Yes | Yes | Yes |
| Harvey | 2021 | The ambulance service advanced practitioner's role in supporting care homes: a qualitative study of care staff experiences | Qualitative research | Yes | Yes |  | Yes | Yes | Yes | Yes | Yes |
| Hughes | 2021 | Community paramedicine home visits: patient perceptions and experiences | Qualitative research | Yes | Yes |  | Yes | Yes | Yes | Yes | Yes |
| Keefe | 2020 | Behavioural Health Emergencies Encountered by Community Paramedics: Lessons from the Field and Opportunities for Skills Advancement. | Qualitative research | Yes | Yes |  | Yes | Yes | Yes | Yes | Yes |
| Lau | 2018 | Qualitative Evaluation of the Coach Training within a Community Paramedicine Care Transitions Intervention. | Qualitative research | Yes | Yes |  | Yes | Yes | Yes | Yes | Yes |
| Leyenaar | 2021 | Paramedics assessing patients with complex comorbidities in community settings: results from the CARPE study. | Qualitative research | Yes | Yes |  | Yes | Yes | Yes | Yes | Yes |
| Martin | 2016 | Consumer perspectives of a community paramedicine program in rural Ontario. | Qualitative research | Yes | Yes |  | Yes | Yes | Yes | Yes | Yes |
| Martin | 2019 | Perspectives from the frontline of two North American community paramedicine programs: an observational, ethnographic study. | Qualitative research | Yes | Yes |  | Yes | Yes | Yes | Yes | No |
| O'Meara | 2015 | Would a prehospital practitioner model improve patient care in rural Australia? | Qualitative research | Yes | Yes |  | Yes | Yes | Yes | Yes | Yes |
| O'Meara | 2016 | Integrating a community paramedicine program with local health, aged care and social services: An observational ethnographic study | Qualitative research | Yes | Yes |  | Yes | Yes | Yes | Yes | Yes |
| Pearson | 2014 | The Evidence for Community Paramedicine in Rural Areas: State and local findings and the role of the state Flex program | Qualitative research | Yes | Yes |  | Yes | Yes | Yes | Yes | Yes |
| Proctor | 2019 | Home visits from paramedic practitioners in general practice: patient perceptions | Qualitative research | Yes | Yes |  | Yes | Yes | Yes | Yes | Yes |
| Ritchie | 2020 | Readiness of Stakeholders to Adopt Community Paramedicine Programs in Tennessee | Qualitative research | Yes | Yes |  | Yes | Yes | Yes | Yes | Yes |
| Schwab-Reese | 2021 | "They're very passionate about making sure that women stay healthy": a qualitative examination of women's experiences participating in a community paramedicine program. | Qualitative research | Yes | Yes |  | Yes | Yes | Yes | Yes | Yes |
| Stirling | 2007 | Engaging rural communities in health care through a paramedic expanded scope of practice. | Qualitative research | Yes | Yes |  | Yes | Yes | Yes | Yes | Yes |
| Whalen | 2018 | The novel role of paramedics in collaborative emergency centres aligns with their professional identity: A qualitative analysis. | Qualitative research | Yes | Yes |  | Yes | Yes | Yes | Yes | Yes |

*MMAT Quality assessment results- Randomised controlled trial*

| **Lead author** | **Year** | **Title** | **Study design** | **Are there clear research questions?** | **Do the collected data allow to address the research questions?** | **Randomised controlled trials** | **Is randomization appropriately performed?** | **Are the groups comparable at baseline?** | **Are there complete outcome data?** | **Are outcome assessors blinded to the intervention provided?** | **Did the participants adhere to the assigned intervention** |
| --- | --- | --- | --- | --- | --- | --- | --- | --- | --- | --- | --- |
| Ashton | 2017 | Conserving Quality of Life through Community Paramedics. | Economic evaluation/ Randomised controlled trial | Yes | Yes |  | Can't tell | Can't tell | Yes | Can't tell | Can't tell |
| Agarwal | 2020 | Evaluation of a community paramedicine health promotion and lifestyle risk assessment program for older adults who live in social housing: a cluster randomized trial. | Randomised controlled trial | Yes | Yes |  | Yes | Yes | Yes | No | Yes |
| Agarwal | 2020 | Reducing 9-1-1 Emergency Medical Service Calls By Implementing A Community Paramedicine Program For Vulnerable Older Adults In Public Housing In Canada: A Multi-Site Cluster Randomized Controlled Trial. | Randomised controlled trial | Yes | Yes |  | Yes | Yes | Yes | No | Yes |
| Dixon | 2008 | Is it cost effective to introduce paramedic practitioners for older people to the ambulance service? Results of a cluster randomised controlled trial | Randomised controlled trial | Yes | Yes |  | Yes | Yes | Yes | No | Yes |
| Mason | 2007 | Effectiveness of paramedic practitioners in attending 999 calls from elderly people in the community: cluster randomised controlled trial. | Randomised controlled trial | Yes | Yes |  | Yes | Yes | Yes | Yes | Yes |
| Mason | 2008 | Safety of paramedics with extended skills | Randomised controlled trial | Yes | Yes |  | Yes | Yes | Yes | No | Yes |
| Shah | 2018 | Improving the ED-to-Home Transition: The Community Paramedic-Delivered Care Transitions Intervention-Preliminary Findings. | Randomised controlled trial | Yes | Yes |  | Yes | Can't tell | Yes | Yes | Can't tell |

*MMAT Quality assessment results- non-randomised studies*

| **Lead author** | **Year** | **Title** | **Study design** | **Non-Randomised studies** | **Are the participants representative of the target population?** | **Are measurements appropriate regarding both the outcome and intervention (or exposure)?** | **Are there complete outcome data?** | **Are the confounders accounted for in the design and analysis?** | **During the study period, is the intervention administered (or exposure occurred) as intended?** |
| --- | --- | --- | --- | --- | --- | --- | --- | --- | --- |
| Abrashkin | 2019 | Community paramedics treat high acuity conditions in the home: a prospective observational study | Prospective observational |  | Yes | Yes | Yes | No | Yes |
| Agarwal | 2017 | Cost-effectiveness analysis of a community paramedicine programme for low-income seniors living in subsidised housing: the community paramedicine at clinic programme (CP@clinic). | Economic evaluation |  | Yes | Yes | Yes | Yes | Yes |
| Agarwal | 2018 | Feasibility of implementing a community cardiovascular health promotion program with paramedics and volunteers in a South Asian population. | Non-randomised experimental study |  | Yes | Yes | Yes | No | Yes |
| Bennett | 2017 | Community Paramedicine Applied in a Rural Community. | Non-randomised experimental study |  | Yes | Yes | Yes | Yes | Yes |
| Bennett | 2020 | Community Paramedicine Applied in a Rural Community | Pre/post-test with a comparison group study design |  | Yes | Yes | Yes | Yes | Yes |
| Quatman-Yates | 2021 | Assessment of Fall-Related Emergency Medical Service Calls and Transports After a Community-Level Fall-Prevention Initiative | Prevalence study |  | Yes | Yes | Yes | Yes | Yes |
| Xie | 2021 | Economic Analysis of Mobile Integrated Health Care Delivered by Emergency Medical Services Paramedic Teams. | Economic evaluation |  | Yes | Yes | Yes | Yes | Yes |

*MMAT Quality assessment results- Quantitative descriptive studies*

| **Lead author** | **Year** | **Title** | **Study design** | **Are there clear research questions?** | **Do the collected data allow to address the research questions?** | **Quantitative descriptive studies** | **Is the sampling strategy relevant to address the research question?** | **Is the sample representative of the target population?** | **Are the measurements appropriate?** | **Is the risk of nonresponse bias low?** | **Is the statistical analysis appropriate to answer the research question?** |
| --- | --- | --- | --- | --- | --- | --- | --- | --- | --- | --- | --- |
| Abrashkin | 2016 | Providing Acute Care at Home: Community Paramedics Enhance an Advanced Illness Management Program-Preliminary Data. | Cohort study | Yes | Yes |  | Yes | Yes | Yes | Yes | Yes |
| Ash | 2020 | Quality of Life for Persons with Chronic Disease Utilizing Mobile Integrated Healthcare | Cohort study | Yes | Yes |  | Yes | Yes | Yes | Yes | Yes |
| Boykin | 2018 | Interprofessional care collaboration for patients with heart failure. | Cohort study | Yes | Yes |  | Yes | Yes | Yes | Yes | Yes |
| Castillo | 2016 | Mobile Integrated Healthcare: Preliminary Experience and Impact Analysis with a Medicare Advantage Population | Cohort study | Yes | Yes |  | Yes | Yes | Yes | Yes | Yes |
| Constantine | 2021 | Implementation of Drive-through Testing for COVID-19 with Community Paramedics | Cohort study | Yes | Yes |  | Yes | Yes | Yes | Can't tell | Yes |
| Counts | 2017 | An Evaluation of the Environmental and Organizational Factors Associated with the Formation of Community Paramedicine Programs | Cross-sectional study | Yes | Yes |  | Yes | Yes | Yes | Yes | Yes |
| Feldman | 2021 | "House Calls" by Mobile Integrated Health Paramedics for Patients with Heart Failure: A Feasibility Study | Cohort study | Yes | Yes |  | Yes | Yes | Yes | Yes | Yes |
| Gingold | 2021 | The effect of a mobile integrated health program on health care cost and utilization. | Cohort study | Yes | Yes |  | Yes | Yes | Yes | Yes | Yes |
| Hanninen | 2020 | Patients Seeking Retreatment after Community Paramedic Assessment and Treatment: Piloting a Community Paramedic Unit Program in Southwest Finland | Retrospective analysis | Yes | Yes |  | Can't tell | Can't tell | Can't tell | Can't tell | Yes |
| Hoyle | 2012 | Introduction of an extended care paramedic model in New Zealand | Cohort study | Yes | Yes |  | Yes | Yes | Yes | Yes | Yes |
| Jensen | 2016 | Impact of a Novel Collaborative Long-Term Care EMS Model: A Before-and-After Cohort Analysis of an Extended Care Paramedic Program | Cohort study | Yes | Yes |  | Yes | Yes | Yes | Yes | Yes |
| Kant | 2018 | Outcomes and provider perspectives on geriatric care by a nurse practitioner-led community paramedicine program. | Case series | Yes | Yes |  | Yes | Yes | Yes | Yes | Yes |
| Knowles | 2010 | An initiative to provide emergency healthcare for older people in the community: the impact on carers. | Cross-sectional study | Yes | Yes |  | Yes | Yes | Yes | Yes | Yes |
| Mason | 2007 | Effectiveness of emergency care practitioners working within existing emergency service models of care | Cross-sectional study | Yes | Yes |  | Yes | Yes | Yes | Yes | Yes |
| Nejtek | 2017 | A pilot mobile integrated healthcare program for frequent utilizers of emergency department services | Cohort study | Yes | Yes |  | Yes | Yes | No | Yes | Yes |
| Nowrouzi-Kia | 2021 | Quality of work life of paramedics practicing community paramedicine in northern Ontario, Canada: a mixed-methods sequential explanatory study | Cross-sectional study | Yes | Yes |  | Yes | Yes | Yes | Yes | Yes |
| Ruest | 2012 | Community health evaluation completed using paramedic service (CHECUPS): design and implementation of a new community based health program | Case report | Yes | Yes |  | Yes | Yes | Yes | Yes | Yes |
| Ruest | 2017 | Evaluating the impact on 911 calls by an in-home programme with a multidisciplinary team | Case series | Yes | Yes |  | Yes | Yes | Yes | Can't tell | Yes |
| Scharf | 2017 | Mobile Integrated Community Health Pilot Program Descriptive Study: Diagnosis Prevalence and Comorbidity among Program Participants | Prevalence study | Yes | Yes |  | Yes | Yes | Yes | Yes | Yes |
| Seidl | 2021 | Development of a Logic Model to Guide Implementation and Evaluation of a Mobile Integrated Health Transitional Care Program. | Retrospective analysis | Yes | Yes |  | Can't tell | Can't tell | Yes | Can't tell | Can't tell |
| Siddle | 2017 | Mobile integrated health to reduce post-discharge acute care visits: A pilot study. | Cohort study | Yes | Yes |  | Yes | Yes | Yes | Yes | Yes |
| Swain | 2012 | Patient satisfaction and outcome using emergency care practitioners in New Zealand. | Cross-sectional study | Yes | Yes |  | Yes | Yes | Yes | Yes | Yes |
| Tangherlini | 2016 | The HOME Team: Evaluating the Effect of an EMS-based Outreach Team to Decrease the Frequency of 911 Use Among High Utilizers of EMS. | Retrospective analysis | Yes | Yes |  | Yes | Yes | Yes | Yes | Yes |
| Widiatmoko | 2008 | Developing a new response to non-urgent emergency calls: evaluation of a nurse and paramedic partnership intervention | Economic evaluation | Yes | Yes |  | Yes | Yes | Yes | Yes | Yes |

*Quality assessment results- mixed methods studies*

| **Lead author** | **Year** | **Title** | **Study design** | **Are there clear research questions?** | **Do the collected data allow to address the research questions?** | **Mixed methods studies** | **Is there an adequate rationale for using a mixed-methods design to address the research question?** | **Are the different components of the study effectively integrated to answer the research question?** | **Are the outputs of the integration of qualitative and quantitative components adequately interpreted?** | **Are divergences and inconsistencies between quantitative and qualitative results adequately addressed?** | **Do the different components of the study adhere to the quality criteria of each tradition of the methods involved?** |
| --- | --- | --- | --- | --- | --- | --- | --- | --- | --- | --- | --- |
| Adio | 2020 | Community Paramedics' Perception of Frequent ED Users and the Community Paramedicine Program: A Mixed-Methods Study. | Mixed Methods | Yes | Yes |  | Yes | Yes | Yes | Yes | Yes |
| Agarwal | 2019 | Effectiveness of a community paramedic-led health assessment and education initiative in a seniors' residence building: the Community Health Assessment Program through Emergency Medical Services (CHAP-EMS). | Mixed Methods | Yes | Yes |  | Yes | Yes | Yes | Yes | Yes |
| Cooper | 2007 | Collaborative practices in unscheduled emergency care: role and impact of the emergency care practitioner--qualitative and summative findings. | Mixed Methods | No | Yes |  | Can't tell | No | No | Can't tell | Can't tell |
| Cooper | 2004 | The emerging role of the emergency care practitioner | Mixed Methods | Yes | Yes |  | Yes | Yes | Yes | Yes | Yes |
| Flint | 2019 | The Systemic Impacts of Integrated Mobile Healthcare in a State-wide Emergency Medical Services System | Mixed methods | Yes | Yes |  | Yes | Yes | Yes | Yes | Yes |
| Leyenaar | 2019 | Relevance of assessment items in community paramedicine home visit programmes: results of a modified Delphi study. | Delphi | Yes | Yes |  | Yes | Yes | Yes | Yes | Yes |
| Leyenaar | 2019 | Examining consensus for a standardised patient assessment in community paramedicine home visits: a RAND/UCLA-modified Delphi Study. | Delphi | Yes | Yes |  | Yes | Yes | Yes | Yes | Yes |
| Lezzoni | 2018 | Early experiences with the Acute Community Care Program in eastern Massachusetts. | Mixed methods | Yes | Yes |  | Yes | Yes | Yes | Can't tell | Can't tell |
| Martin-Misener | 2009 | Cost effectiveness and outcomes of a nurse, practitioner paramedic, family physician model of care: the Long and Brier Islands study | Economic evaluation | Yes | No |  | Can't tell | Yes | Yes | No | Can't tell |
| Misra-Hebert | 2021 | Healthcare utilization and patient and provider experience with a home visit program for patients discharged from the hospital at high risk for readmission. | Mixed Methods | Yes | Yes |  | Yes | Yes | Yes | Yes | Yes |

*MMAT Quality assessment results- Studies not included in MMAT*

| **Lead author** | **Year** | **Title** | **Study design** | **Are there clear research questions?** | **Do the collected data allow to address the research questions?** | **Comments** |
| --- | --- | --- | --- | --- | --- | --- |
| Ball | 2005 | Setting the scene for the paramedic in primary care: a review of the literature. | Literature review | Yes | Yes | No MMAT category available- no significant risk of bias noted to warrant exclusion |
| Batt | 2021 | Advances in Community Paramedicine in Response to COVID-19 | Grey literature | Yes | Yes | No MMAT category available- no significant risk of bias noted to warrant exclusion |
| Bigham | 2013 | Expanding paramedic scope of practice in the community: a systematic review of the literature. | Systematic review | Yes | Yes | No MMAT category available- no significant risk of bias noted to warrant exclusion |
| Bradley | 2016 | The business case for community paramedicine: lessons from Commonwealth Care Alliances Pilot Program | Grey literature | No | Yes | No MMAT category available- no significant risk of bias noted to warrant exclusion |
| Chan | 2019 | Community paramedicine: A systematic review of program descriptions and training. | Systematic review | Yes | Yes | No MMAT category available- no significant risk of bias noted to warrant exclusion |
| Choi | 2016 | Mobile Integrated Health Care and Community Paramedicine: An Emerging Emergency Medical Services Concept. | Systematic review | No | Yes | No MMAT category available- no significant risk of bias noted to warrant exclusion |
| Eaton | 2021 | Designing and implementing an educational framework for advanced paramedic practitioners rotating into primary care in North Wales | Literature review | Yes | Yes | No MMAT category available- no significant risk of bias noted to warrant exclusion |
| Eaton | 2020 | Understanding the role of the paramedic in primary care: a realist review. | Literature review | Yes | Yes | No MMAT category available- no significant risk of bias noted to warrant exclusion |
| Eaton | 2021 | Contribution of paramedics in primary and urgent care: a systematic review. | Systematic review | Yes | Yes | No MMAT category available- no significant risk of bias noted to warrant exclusion |
| Evans | 2012 | Which extended paramedic skills are making an impact in emergency care and can be related to the UK paramedic system? A systematic review of the literature. | Systematic review | Yes | Yes | No MMAT category available- no significant risk of bias noted to warrant exclusion |
| Glenn | 2017 | State Regulation of Community Paramedicine Programs: A National Analysis. | Systematic review | Yes | Yes | No MMAT category available- no significant risk of bias noted to warrant exclusion |
| Goldberg | 2014 | Mobile integrated healthcare: Using existing out of hospital resources to bridge gaps in healthcare services | Systematic review | Yes | Yes | No MMAT category available- no significant risk of bias noted to warrant exclusion |
| Gregg | 2019 | Systematic Review of Community Paramedicine and EMS Mobile Integrated Health Care Interventions in the United States. | Systematic review | Yes | Yes | No MMAT category available- no significant risk of bias noted to warrant exclusion |
| Halter | 2006 | Patients' experiences of care provided by emergency care practitioners and traditional ambulance practitioners: A survey from the London Ambulance Service | Survey | Yes | Yes | No MMAT category available- no significant risk of bias noted to warrant exclusion |
| Hill | 2013 | A systematic review of the activity and impact of emergency care practitioners in the NHS. | Systematic review | Yes | Yes | No MMAT category available- no significant risk of bias noted to warrant exclusion |
| Leduc | 2020 | The Safety and Effectiveness of On-Site Paramedic and Allied Health Treatment Interventions Targeting the Reduction of Emergency Department Visits by Long-Term Care Patients: Systematic Review. | Systematic review | Yes | Yes | No MMAT category available- no significant risk of bias noted to warrant exclusion |
| Leyenaar | 2021 | What do community paramedics assess? An environmental scan and content analysis of patient assessment in community paramedicine. | Environmental scan | Yes | Yes | No MMAT category available- no significant risk of bias noted to warrant exclusion |
| Leyenaar | 2019 | Report on the status of community paramedicine in Ontario | Grey literature | No | Yes | No MMAT category available- no significant risk of bias noted to warrant exclusion |
| Leyenaar | 2018 | A scoping study and qualitative assessment of care planning and case management in community paramedicine | Grey literature | Yes | Yes | No MMAT category available- no significant risk of bias noted to warrant exclusion |
| O'Meara | 2003 | Community paramedicine model of care: an observational, ethnographic case study. | Soft systems methodology | Yes | Yes | No MMAT category available- no significant risk of bias noted to warrant exclusion |
| Pang | 2019 | Limited data to support improved outcomes after community paramedicine intervention: A systematic review. | Systematic review | Yes | Yes | No MMAT category available- no significant risk of bias noted to warrant exclusion |
| Pearson | 2017 | Community Paramedicine pilot programs: lessons from Maine | Survey exercise and literature review | Yes | Yes | No MMAT category available- no significant risk of bias noted to warrant exclusion |
| Rasku | 2019 | The core components of Community Paramedicine - integrated care in primary care setting: a scoping review. | Systematic review | Yes | Yes | No MMAT category available- no significant risk of bias noted to warrant exclusion |
| Raynovich | 2014 | A survey of community paramedicine course offerings and planned offerings | Survey | Yes | Yes | No MMAT category available- no significant risk of bias noted to warrant exclusion |
| Thirumalai | 2021 | Challenges and Lessons Learned from a Telehealth Community Paramedicine Program for the Prevention of Hypoglycaemia: Pre-Post Pilot Feasibility Study | Single-arm pre/post-test | Yes | Yes | No MMAT category available- no significant risk of bias noted to warrant exclusion |
| Thompson | 2014 | HWA Expanded Scopes of Practice program evaluation: Extending the Role of Paramedics sub-project | Grey literature | No | Yes | No MMAT category available- no significant risk of bias noted to warrant exclusion |
| Thurman | 2021 | A scoping review of community paramedicine: evidence and implications for interprofessional practice. | Scoping review | Yes | Yes | No MMAT category available- no significant risk of bias noted to warrant exclusion |
| Tohira | 2013 | The impact of new prehospital practitioners on ambulance transportation to the emergency department: a systematic review and meta-analysis. | Systematic review | Yes | Yes | No MMAT category available- no significant risk of bias noted to warrant exclusion |
| van Vuuren | 2021 | Reshaping healthcare delivery for elderly patients: the role of community paramedicine; a systematic review. | Systematic review | Yes | Yes | No MMAT category available- no significant risk of bias noted to warrant exclusion |
| Xi | 2021 | Paramedics working in general practice: a scoping review | Scoping review | Yes | Yes | No MMAT category available- no significant risk of bias noted to warrant exclusion |
